# An Integrated Fecal Microbiome and Metabolomics in T2DM Rats Reveal Antidiabetes Effects from Host-Microbial Metabolic Axis of EtOAc Extract from *Sophora flavescens*

**DOI:** 10.1155/2020/1805418

**Published:** 2020-05-27

**Authors:** Jing Shao, Yi Liu, Huan Wang, Yun Luo, Lei Chen

**Affiliations:** ^1^Key Laboratory of Digital Quality Evaluation of Chinese Materia Medical of State Administration of TCM, China; ^2^Engineering & Technology Research Centre for Chinese Materia Medical Quality of Guangdong Province, School of Traditional Chinese Medicine, Guangdong Pharmaceutical University, Guangzhou 510006, China; ^3^School of Chinese Medicine, Southern Medical University, Guangzhou 510515, China

## Abstract

Type 2 diabetes mellitus (T2DM) is a chronic metabolic disease. *Sophora flavescens* (*S. flavescens*), also named Kushen, is a famous Chinese herbal medicine that has been used to prevent and cure T2DM both in folk medicine and in medical institution. However, its mechanism of action remains unclear. In this study, the pharmacodynamic effects of *S. flavescens* EtOAc extract (SFE) on high-fat diet and low-dose streptozotocin-induced T2DM rats were examined. Fecal metabolomics analysis and 16S rRNA gene sequencing were applied to determine the influence of T2DM and SFE treatment on gut microbiota and host metabolism. Based on the consistency of the results of metabolic pathways in metabolomics analysis and phylogenetic investigation of communities by reconstruction of unobserved state (PICRUSt) analysis of 16S rRNA gene sequencing, the level of metabolites and the operational taxonomic units of gut bacteria were combined, and Spearman's analysis was implemented. Our data showed that SFE significantly decreased fasted blood glucose levels and improved lipid profile, glycosylated serum protein, glycosylated hemoglobin index, and pancreas damage. Metabolomics and 16S rRNA gene sequencing analysis indicated gut bacteria disorder, disturbed lipid metabolism, carbohydrate metabolism, and especially amino acid metabolism in T2DM and that SFE can regulated these metabolic pathways through the influence on gut bacteria. Spearman's analysis indicated that the amino acid metabolism that included tryptophan, branched chain amino acid, aromatic amino acid, beta-alanine, and glycine, serine and threonine metabolism, lipid metabolism, including lysophosphatidylcholines and lysophosphatidylethanolamines, primary bile acid and linoleic acid metabolism, carbohydrate metabolism, and nucleotide metabolism positively correlated with *Faecalibacterium*, *Flexispira*, *Phascolarctobacterium*, *Prevotella*, *Roseburia*, and *[Prevotella]*. In addition, arginine and proline metabolism, steroid hormone, steroid biosynthesis, and sphingolipid metabolism positively correlated with *Lactobacillus*, *Oscillospira*, *Parabacteroides*, *Ruminococcus*, and *Streptococcus*. Taken together, we speculated that SFE may have an effect on T2DM by mediating host-microbial metabolic axis. Exploration of SFE treatment for T2DM by multiomics is expected to provide a reference for clinical treatment.

## 1. Introduction

Type 2 diabetes mellitus (T2DM) is a dramatic chronic metabolic disease that is characterized by a high blood glucose level and has a high prevalence worldwide [1]. Oxidative stress can lead to the development of diabetes by damaging islet *β* cells and reducing the sensitivity of peripheral tissues to insulin. Persistent hyperglycemia and oxidative stress can result in multiorgan damage and complications, and T2DM becomes a major threat to human life and health after cardiovascular diseases and cancer [2].

The human intestinal tract is a natural bioreactor for diverse and highly mutualistic microorganisms and accommodates a variety of gut microbiota, which are important for glucose, amino acid, and lipid metabolism [3, 4]. Gut bacteria can directly affect host metabolism through fermentation processes and the production of bioactive metabolites and regulate the process of metabolic diseases. Therefore, elucidating the regulation of the host-microbial metabolic axis is of utmost importance [5]. Significant evidence has indicated an altered microbial composition in T2DM patients [6–9]. Gut bacteria have been used as an important target for the prevention and treatment of T2DM, and studies on the treatment of T2DM by transplantation of gut bacteria in healthy hosts and probiotics have long been reported [3]. However, the contribution of gut bacterial metabolism to the host is not clear and a systematic description of it is not available. Exploration of the role of gut bacteria is particularly crucial to T2DM treatment.

“Kushen,” a well-known traditional Chinese medicinal herb stemmed from the dried roots of *Sophora flavescens* (*S. flavescens*), has been used in the counteraction of T2DM in folk medicine and medical institution in southern Fujian and south of the Five Ridges, China [10, 11]. Modern pharmacological studies have shown that flavonoids derived from Kushen exhibited antidiabetic activity. However, most studies have focused on the role of flavonoids in Kushen at the cellular or target protein level, and studies that focused on evaluating the antidiabetic effects evaluation based on the overall animal are lacking [12–15]. Recently, it was reported that flavonoid compounds derived from *S. flavescens* EtOAc extract (SFE) influence the treatment of T2DM in KK-Ay mice [16]; however, studies on identifying the treatment mechanism are lacking. Moreover, the results obtained from a spontaneous diabetic mouse model are still to be validated in animal models that are more in line with human pathogenesis and pathological processes.

**Figure 1:**
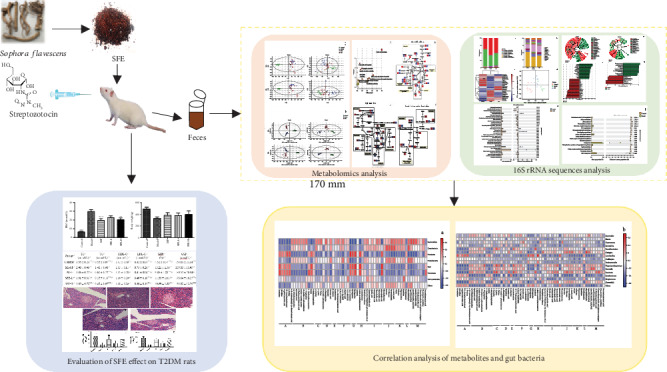
The strategy of investigating the mechanism of SFE treating T2DM through the host-microbial metabolic axis by integrated fecal microbiome and metabolomics approaches.

Feces samples can reflect intestine-related metabolites better, and to better understand the role of intestinal flora metabolism in T2DM and SFE treatment, 16S rRNA gene sequencing analysis was used to explore the changes in the structure of gut bacteria and the discriminative bacteria under T2DM and SFE treatment; phylogenetic investigation of communities by reconstruction of unobserved state (PICRUSt) of 16S rRNA gene sequencing analysis was used to predict the metabolic function of microbiota [17–20]. And fecal metabolomics was performed to identify biomarkers related to intestinal flora metabolism. Finally, Spearman's analysis between the level of metabolites and the operational taxonomic units (OTUs) of gut bacteria was performed to elucidate the regulatory relationship between gut bacteria and metabolites. The strategy of our study is presented in Figure 1. In this study, an integrated fecal microbiome and metabolomics were used to elucidate the mechanism of SFE on treating T2DM and how gut bacteria regulate the development of T2DM through the host-microbial metabolic axis.

## 2. Materials and Methods

### 2.1. Instruments and Reagents

*S. flavescens* samples were purchased from the planting base of Radix *Sophora* GAP in Chinese medicinal materials from Shanxi Zhendong Pharmaceutical Co., Ltd. (Shanxi, China). Ethyl acetate and ethanol were obtained from Guangzhou Huaxin Chemical Reagent Co., Ltd. (Guangzhou, China). Distilled water was produced by China Watsons Food & Beverage Co., Ltd. (Hong Kong, China). Chromatographic grade acetonitrile and formic acid were purchased from Fisher Scientific Co., Ltd. (Cambridge, MA, USA). Citric acid, sodium citrate, and chloral hydrate were obtained from China Tianjin Zhiyuan Co., Ltd. (Tianjin, China). Streptozotocin (STZ) was produced by American Sigma Co., Ltd. (St. Louis, MO, USA). Paraformaldehyde was purchased from Yantai Shuangshuang Chemical Co., Ltd. (Shandong, China). The cholesterol kit was purchased from Zhongsheng North Control Biotechnology Co., Ltd. (Beijing, China). Glycosylated serum protein kit, low-density lipoprotein cholesterol (LDL-C) test kit, and high-density lipoprotein cholesterol (HDL-C) test kit were purchased from Nanjing Construction Co., Ltd. (Jiangsu, China). MOBIO PowerSoil® DNA Isolation Kit was purchased from MOBIO Laboratories (Carlsbad, CA, USA). NanoDrop One was obtained from Fisher Scientific Co., Ltd. (Cambridge, MA, USA). EZNA Gel Extraction Kit was brought from Omega Bio-Tek Co., Ltd. (Norcross, GA, USA).

The blood glucose tester was obtained from Johnson & Johnson Co., Ltd. (Piscataway, NJ, USA). The AU400 automatic biochemical analyzer was manufactured by Olympus Corporation Co., Ltd. (Tokyo, Japan). BioRad S1000 was produced by Bio-Rad Laboratory (Irvine, CA, USA). The Eclipse E100 light microscope was manufactured by Nikon Corporation Co., Ltd. (Tokyo, Japan).

### 2.2. Preparation and Determination of SFE

*S. flavescens* samples were identified by Dr. Lei Chen of the Guangdong Pharmaceutical University. Referring to the preparation method determined in our previous study [21], slices of *S. flavescens* were extracted 3 times for 2 hours by reflux extraction using 90% ethanol. Combined extracts were evaporated, and the residue was reconstituted in warm water (1 : 8, *v*/*v*), and filtered. The aqueous solution was further extracted four times with ethyl acetate (1 : 1, *v*/*v*). After removal of ethyl acetate by a rotary evaporator, SFE was obtained by lyophilization.

Quantification of total flavonoid compounds in SFE was carried out by UV spectrophotometry as by Zhu [22]. In brief, a batch of SFE with 3 samples was weighted, and its contents were determined according to the detailed step in Supplementary Materials (Supplementary Materials: 1.2. The quantification of total flavonoid compounds in SFE). Additionally, according to our previous study [21], the content determination of 4 flavonoid compounds in SFE was performed on a UHPLC-MS system, and the flavonoid compounds in SFE were eluted gradient by ACQUITY BEH C_18_ (2.1 × 100 mm, particle size 1.7 *μ*m; Waters, MA, USA). The conditions of UHPLC-MS analysis are presented in the Supplementary Materials (Supplementary Materials: 1.3. The conditions of UHPLC-MS analysis of 4 flavonoid compounds in SFE).

### 2.3. Animal Experiments

#### 2.3.1. Animal Grouping

All procedures involving animals were conducted according to the guidelines of the National Health Institutes of China and were approved by the animal ethics committee of Guangdong Pharmaceutical University (Guangzhou, China). Male Sprague-Dawley (SD) rats aged 8 to 10 weeks and weighting 180-220 g were supplied by the lab animal center of Southern Medical University (Guangzhou, China, quality certificate number: SYXK2012-0125, batch number: 44002100008034). Animals were housed in a 22 ± 3°C animal room with a 50 ± 10% humidity and maintained on a 12-hour light-dark cycle. During acclimation, rats were fed a normal diet and had access to water ad libitum.

Except for the control group, rats were fed a high-fat diet (HFD, 70% normal chow diet, 9% lard, 1% cholesterol, 15% sucrose, 5% yolk powder; purchased from Guangdong Medical Animal Center) for 4 consecutive weeks, starting after the week of acclimation. After 4 weeks, rats that were fed a HFD were intraperitoneally injected with streptozotocin (STZ, 35 mg/kg body weight (BW)) after being fasted for 12 hours to induce T2DM. Control rats received an equivalent volume of sterile saline. Rats injected with STZ that had a fasted blood glucose (FBG) higher than 16.7 mmol/L were diagnosed with T2DM. Subsequently, 48 T2DM rats were randomly selected and divided into 4 groups. These groups include the model group, metformin (MF) group, and low-dose (SFE-L 37.5 mg/kg/day), and high-dose (SFE-H 75 mg/kg/day) SFE groups. The dose of *S. flavescens* as recommended by the Chinese Pharmacopoeia is 4.5-9 g of raw herb per day. Using a conversion based on the body surface-area ratio of rats and humans and the extraction rate of SFE, the daily treatment dose for rats was calculated as 37.5 mg/kg and 75 mg/kg (SFE).

#### 2.3.2. Collection of Feces Samples

Two hours after administration of SFE on the last day of the fourth and eighth weeks, rat feces within 12 hours were collected using a specific pathogen-free (SPF) animal feeding platform. Rats were placed in sterilized metabolism cages (one rat per cage), and feces were collected on ice and placed equally in two sterile microcentrifuge tubes. Samples were stored at -80°C until analysis. According to pharmacodynamic results, the high-dose SFE group has shown the best treatment effect; therefore, feces samples from the control, model, and high-dose SFE groups of the fourth and eighth weeks were used for fecal metabolomics analysis, and feces samples of the eighth week were used for 16S rRNA gene sequencing analysis.

### 2.4. Evaluation of SFE Effect on T2DM Rats

The BW of rats was measured every 3 days, and FBG was weekly measured using an automatic glucometer by the tail vein pricking method after 8 hours of fasting. Serum total cholesterol (TC), serum triglycerides (TG), serum high-density lipoprotein cholesterol (HDL-C), serum low-density lipoprotein cholesterol (LDL-C), glycosylated serum protein (GSP), and glycosylated hemoglobin (GHb) were determined by an automatic biochemical analyzer. After 8 weeks of treatment, rats were euthanized and the pancreas from each rat was isolated and fixed in 4% paraformaldehyde for 48 hours, then dehydrated, permeated, embedded in wax, and sliced into sections. Sections were stained with hematoxylin and eosin. The pathological observation of the pancreas was performed under a light microscope with 200x magnification. Under the 20x magnification, the number of islets in each visual field was measured, and the total number of islets in 5 visual fields was counted; 5 islets were randomly selected for each section, the area of each islet was measured with a micrometer, and the total area of them was counted [23].

### 2.5. 16S rRNA Gene Sequencing Analysis

The treatment process of feces used for 16S rRNA gene sequencing analysis was as follows: the MOBIO PowerSoil® DNA Isolation Kit was utilized to extract microbial DNA from feces samples. The concentration and purity were determined using NanoDrop One. 16S rRNA genes of distinct regions (16S: V4-V5) were amplified using a specific primer with 12 bp barcode. Primers were synthesized by Invitrogen (Carlsbad, CA, USA). PCR reactions, containing 25 *μ*L 2x Premix Taq (Takara Biotechnology, Dalian Co. Ltd., Dalian, China), 1 *μ*L each primer (10 mM) and 3 *μ*L DNA (20 ng/*μ*L) template in a total volume of 50 *μ*L, were amplified by thermocycling as follows: 5 min at 94°C for initialization; 30 cycles of 30 s denaturation at 94°C, 30 s annealing at 52°C, and 30 s extension at 72°C, followed by 10 min final elongation at 72°C. The PCR instrument was a BioRad S1000. The length and concentration of the PCR product were determined by 1% agarose gel electrophoresis. Samples with the bright main strip between 400 and 450 bp can be used for further experiments. PCR products were mixed in equidensity ratios according to GeneTools Analysis Software (version 4.03.05.0, SynGene). Then, mixed PCR products were purified with the EZNA Gel Extraction Kit. Each project selects the appropriate primers for amplification. Sequencing libraries were generated using NEBNext® Ultra™ DNA Library Prep Kit for Illumina® (New England Biolabs, MA, America) following the manufacturer's recommendations, and index codes were added. The library quality was assessed on the Qubit@ 2.0 Fluorometer (Thermo Fisher Scientific, Waltham, MA, USA) and Agilent Bioanalyzer 2100 system (Agilent Technologies, Waldbronn, Germany). At last, the library was sequenced on an IlluminaHiseq2500 platform and 250 bp paired-end reads were generated.

16S rRNA gene sequencing analysis was performed using usearch software (V8.0.1517, http://www.drive5.com/usearch/). OTUs were generated from sequences with at least 97% similarity. First, an OTU is thought to possibly represent a species. The most frequently occurring sequence was extracted as a representative sequence for each OTU and was screened for further annotation. Based on the relative abundance of species at each classification level in otu_table, R software was used to draw the histogram and heat map. Second, alpha diversity was applied for analyzing the complexity of species diversity of a sample through 5 indices, including Observed species, Chao1, Shannon, Simpson, and Dominance. In our samples, these indices were calculated using QIIME (http://qiime.org/, V1.9.1) and displayed using R software (V2.15.3). Two indices were selected to identify community richness: Observed species and Chao1, and three indices were used to identify community diversity: Shannon, Simpson, and Dominance. For more information on the diversity index, see http://www.mothur.org/wiki/Calculators, http://scikit-bio.org/docs/latest/generated/skbio.diversity.alpha.html. Third, beta diversity analysis was used to evaluate differences between samples of species complexity. Bray-Curtis weighted and unweighted unifrac beta diversity indexes were calculated using QIIME software. Principal Coordinate Analysis (PCoA) was performed to obtain principal coordinates and visualize from complex, multidimensional data. A distance matrix of weighted or unweighted unifrac among samples obtained before was transformed to a new set of orthogonal axes, by which the maximum variation factor was demonstrated by the first principal coordinate, the second maximum by the second principal coordinate. PCoA was displayed by the qiime2 and ggplot2 package in R software. Fourth, statistical analysis of differences between groups was carried out by Linear discriminant analysis (LDA) Effect Size (LEfSe) analyses. Significantly different gut bacteria can be selected among the three groups. Finally, the functions of gut bacteria were predicted utilizing PICRUSt (http://picrust.github.io/picrust/), which was used to identify the OTU involved in the metabolic pathways based on the 16S rRNA data set.

### 2.6. Fecal Metabolomics Analysis

The treatment process of feces samples used for fecal metabolomics analysis was as follows: feces samples were taken out at -80°C and placed in a freeze dryer for drying. A total of 50 mg feces from each sample was place in a 2 mL sterile tube. After screening and optimization, 300 *μ*L precooled acetonitrile and 100 *μ*L precooled distilled water that acted as extraction solvent were added to each sample. Then, the mixture was vigorously vortexed for 3 min and sonicated for 5 min. After blending and centrifuging at 15,000 rpm for 15 min at 4°C, the supernatant was obtained, and 10 *μ*L of supernatant from each sample was mixed to obtain a quality control (QC) sample. Samples were filtered through a 0.22 *μ*m microporous membrane for UHPLC-MS analysis.

The chromatographic separation system consisted of a C_18_ column (2.1 mm × 100 mm, 1.7 *μ*m, Waters, MA, USA) and a guard cartridge (2.1 mm × 5 mm, 1.7 *μ*m, Waters, MA, USA) at 30°C. The autosampler was set at 4°C. Detection of mass spectrometry information of metabolites was achieved on an Ultimate 3000 LC system coupled to a Quadrupole-Exactive Orbitrap-Mass Spectrometry with an ESI source (Thermo Fisher Scientific, Karlsruhe, Germany). Mass Spectrometry conditions were as follows: electrospray voltage of 3.0 kV, capillary temperature of 320°C, sheath gas flow rate of 15 L/min, the auxiliary gas flow rate of 5 L/min, and collision energy of 15, 35, and 55%. The scanning range was 50-750 (*m*/*z*). Prior to the analysis of samples, the QC sample was run 6 times to balance the system and was run once every 6 samples during the run to check the stability of the system. The mobile phase consisted of water (A) and acetonitrile (B), each containing 0.1% formic acid. The gradient program was as follows: 5% B from 0 to 1.5 min, 50% B from 1.5 to 3 min, 55% B from 3 to 7.5 min, 95% B from 7.5 to 12 min, and 95% B from 12 to 15 min. The flow rate was 0.3 mL/min, and the injection volume was 3 *μ*L.

UHPLC-MS raw data were analyzed by Compound Discoverer 3.0 software (Thermo Fisher Scientific, MA, USA). The peaks of the original data were identified and matched to obtain data consisting of sample number, retention time, molecular weight, and peak area. The data matrix was analyzed by principal component analysis (PCA) and orthogonal partial least-squared discriminant analysis (OPLS-DA) using SIMCA-P 14.0 software (https://umetrics.com/products/simca). To evaluate the model, the *R*^2^*X*, *R*^2^*Y*, and *Q*^2^ parameters were used. In the score map generated by OPLS-DA analysis, the variable importance in projection (VIP) value > 1 and fold change (FC) values more than 2 and less than 0.5 were considered to have an effect on the separation between groups, and these candidates were selected for significance testing. Before the significance test, the data normal distribution and homogeneity test of variance analysis were carried out. The peak area of these candidates underwent a normal standardization procedure to prepare the following analysis: the Shapiro-Wilk test was used to investigate normal distribution, and the Levene test was used to investigate homogeneity of variance. If the data followed a normal distribution and showed equal variance, Student *t*-test was performed. If the data followed a normal distribution but did not show equal variance, Welch's *t*-test was employed. If the data did not follow a normal distribution, the nonparametric test was performed. Candidates with a significant test of *p* < 0.05 were chosen as potential biomarkers. In group-to-group comparisons, FC values of potential biomarkers greater than 2 were considered upregulated whereas FC values of less than 0.5 were considered downregulated. The identification of biomarkers was mainly based on their accurate molecular mass and secondary mass spectrometry information, and the information of these biomarkers was matched with literature reports, database Metlin (http://metlin.scripps.edu), and Human Metabolome Database (HMDB) (http://www.hmdb.ca/). The identified biomarkers were analyzed by MetaboAnalyst 4.0 (http://www.MetaboAnalyst.ca/) in conjunction with the Kyoto Encyclopedia of Genes and Genomes (KEGG) database (http://www.genome.jp/kegg) to analyze relevant metabolic pathways.

### 2.7. Correlation Analysis of Metabolites and Gut Bacteria

Correlation analysis of the levels of metabolites (potential biomarkers) and OTUs of gut bacteria was performed by omicsolution (https://www.omicsolution.org/wkomics/main), and R software (R version 3.6.0 https://www.r-project.org/) was used to visualize their correlation coefficient that was obtained from omicsolution, and their relationship was displayed by a heat map.

### 2.8. Statistical Analysis

The FBG, BW, biochemical indexes, and number and area of islets were analyzed by the one-way ANOVA test, and the results were expressed as the mean ± SD. In 16S rRNA analysis, the *K*-Sample Fisher-Pitman Permutation test was used for the difference analysis of alpha diversity among the control, T2DM, and SFE groups. At Linear discriminant analysis (LDA) Effect Size (LEfSe) analyses, first, the nonparametric factorial Kruskal-Wallis (KW) sum-rank test was used to detect the species with significant abundance differences between groups; second, the Wilcoxon rank sum test was used to analyze differences between two groups. Statistical Analysis of Metagenomic Profiles (STAMP, http://kiwi.cs.dal.ca/Software/STAMP) was used to perform Welch's *t*-test to analyze the predicted functions of gut bacteria. Spearman's correlation analysis was used to calculate the correlation coefficient between metabolites and gut bacteria.

## 3. Results

### 3.1. The Content of Flavonoid Compounds in SFE

SFE was prepared, and UV spectrophotometry was used to determine the content of total flavonoid compounds. The average content of total flavonoid in three parallel measurements of SFE was 61.32%, and the relative standard deviation (RSD) was 2.18%.

The flavonoid compounds in SFE were unambiguously identified by comparing the retention time and fragment ions in the MS^2^ spectra with the reference standards. Based on the total ion chromatogram (TIC) in the negative ion mode, twenty-seven flavonoid compounds were identified (Supplementary Materials: Figure S1), and information of the compound is presented in Supplementary Materials (Table S1). Moreover, four flavonoid compounds in SFE were quantitatively determined, and the contents of kurarinone, norkurarinone, kushenol N, and kushenol L in SFE were 63.2, 35.3, 23.7, and 1.9 mg/g, respectively [21].

### 3.2. The Treatment Effect of SFE on T2DM Rats

#### 3.2.1. SFE Effects on FBG and BW

**Figure 2:**
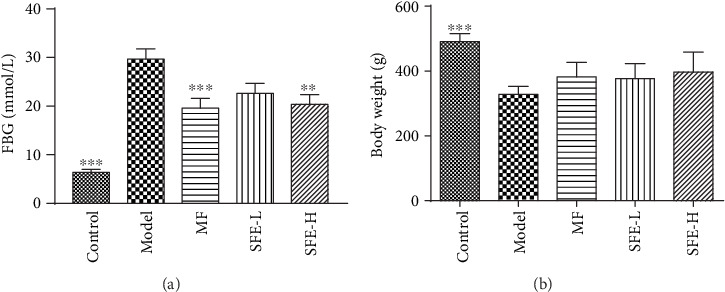
Effects of SFE on FBG levels (a) and BW (b) after 8 weeks of treatment (*n* = 12). ∗ indicates a significant difference compared with the model group: ^∗∗^*p* < 0.01; ^∗∗∗^*p* < 0.001.

As shown in Figure 2(a), after eight weeks of treatment, the FBG levels of each rat were measured. The model rats showed a high FBG level, and the FBG levels of rats treated with a high dose of SFE significantly decreased when compared with the model group (*p* < 0.01); however the FBG levels were still higher when compared to that of rats in the control group. Rats treated with a low dose of SFE also showed lower FBG levels when compared to model group rats; however, the decrease in FBG levels was not statistically significant. On the other hand, during the experiment, the BW of control rats continued to increase, whereas model rats showed gradual emaciation at the end of the experiment (*p* < 0.001) when compared with control rats, and the BW of treated model rats showed a slowly increasing trend (Figure 2(b)). The above results indicated that SFE has an inhibitory effect on high FBG levels in model rats and SFE increased the BW of model rats.

#### 3.2.2. SFE Effects on Biochemical Indexes

In this study, the effects of SFE on TC, TG, HDL-C, LDL-C, GSP, and GHb concentrations in model rats were investigated. As shown in Table 1, when compared with those in the control group, rats in the model group showed an increase in plasma TC, TG, LDL-C, GHb, and GSP and a decrease in HDL-C. A high dose of SFE significantly decreased the levels of TC, TG, LDL-C, GHb, and GSP, thereby showing the best treatment effect. HDL-C was increased by SFE treatment; however, the effect was not significant.

#### 3.2.3. SFE Effects on Pancreas Histomorphological Changes

**Figure 3:**
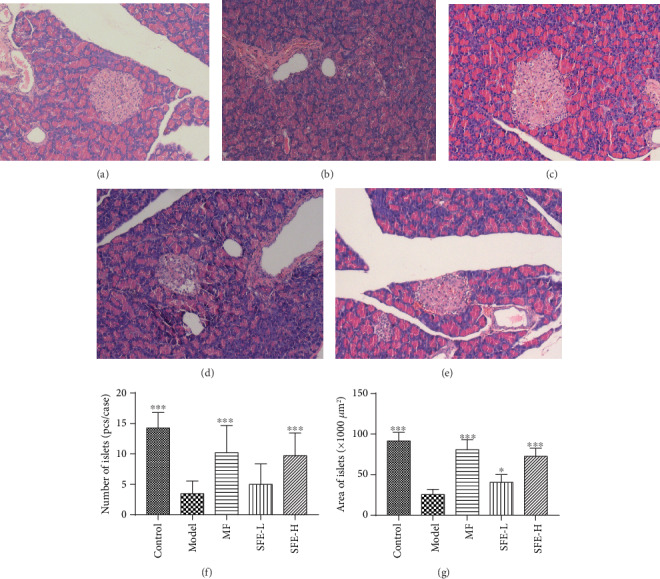
Effects of SFE on the histomorphological changes of the pancreas. The pathological observation (optic microscopy: 200x) of the (a) control group, (b) model group, (c) MF group, (d) SFE treated group (37.5 mg/kg), and (e) SFE-treated group (75 mg/kg). (f) The number and (g) area of islets of all groups (*n* = 12). ^∗^Significant difference compared with the model group: ^∗^*p* < 0.05, ^∗∗^*p* < 0.01, ^∗∗∗^*p* < 0.001.

Histological examination of the pancreas (Figures 3(a)–3(e)) showed that the pancreatic islets of control rats were round with oval cell masses and were highly abundant in the cytoplasm, regular in shape, and clear in the boundary. With the development of the T2DM, islets of the pancreas became seriously damaged, the pancreatic islet cells were atrophic, the number of islet cells was reduced, the cytoplasm was completely atrophic, and the cell boundaries were unclear. After 8 weeks of SFE treatment, pathological damage was greatly alleviated, and the abnormity structure of the pancreases was ameliorated, especially in rats that were treated with a high dose of SFE. The pancreas of rats treated with a low dose of SFE was slightly improved. The number and area of islets in the model group were significantly lower than that in control group and were increased significantly after being treated with a high dose of SFE. Although the number of islet cells in low-dose SFE-treated rats was not significantly increased, the decrease of the islet area was improved (Figures 3(e) and 3(f)).

### 3.3. The Result of 16S rRNA Gene Sequencing Analysis

#### 3.3.1. Response of Gut Bacteria Structure to the SFE in T2DM Rats

Rarefaction analysis showed that the curve in each group reached a plateau, which demonstrated an adequate sequencing depth of all samples (Supplementary Materials: Figure S2a, b). As shown in alpha diversity analysis, rats in the model group showed less abundance when compared with the rats in the control group, and SFE greatly increased the richness of gut bacteria (Supplementary Materials: Figure S2c, d).

**Figure 4:**
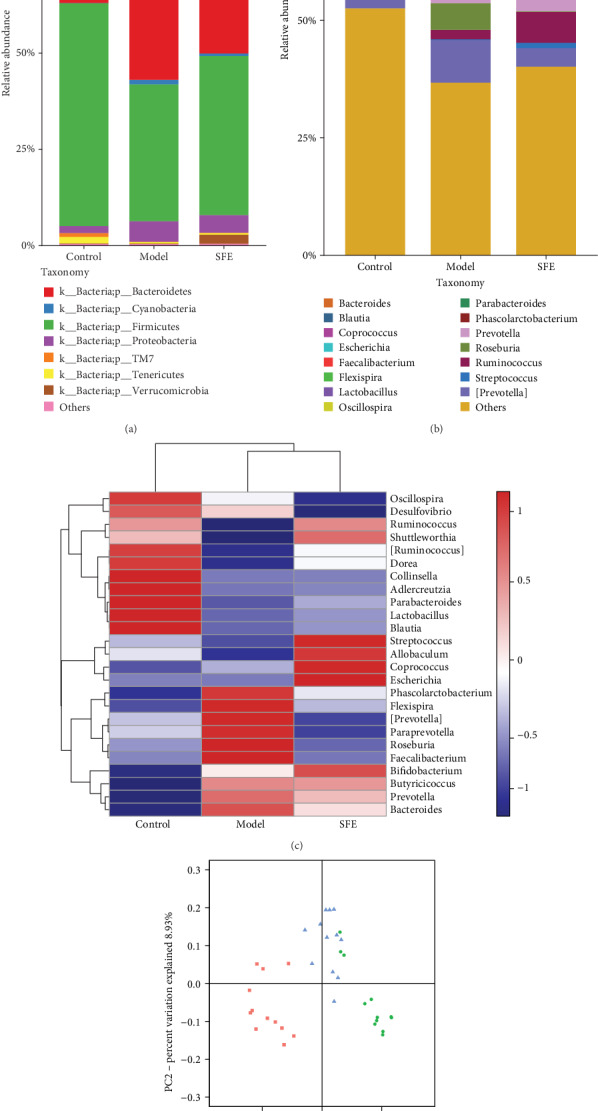
SFE administration altered the gut microbiota structure in T2DM rats. Bacteria composition of the different communities at the (a) phylum level and (b) genus level in the control, T2DM, and SFE groups in the eighth week. (c) The community heat map of the control, T2DM, and SFE groups in the eighth week. (d) The principal co-ordinate analysis among the control, T2DM, and SFE groups based on Unweighted_Unifrac in the eighth week.

The composition of gut microbiome at the phylum and genus levels is presented in Figures 4(a) and 4(b). At the phylum level, *Bacteroidetes*, *Firmicutes*, and *Proteobacteria* make up a large part; *Bacteroidetes* and *Proteobacteria* were increased in model rats when compared with control rats, while *Firmicutes* was decreased. At the genus level, the reduced abundance of *Blautia*, *Clostridium*, *Escherichia*, *Faecalibacterium*, *Lactobacillus*, *Parabacteroides*, *Ruminococcus*, *Oscillospira*, and *Streptococcus* and the increased abundance of *Bacteroides*, *Flexispira*, *Phascolarctobacterium*, *Prevotella*, *Roseburia*, *[Prevotella]*, and *Coprococcus* were observed in model rats. After treatment with SFE, the structure of bacteria in model rats tended to control rats.

From the community heat map diagram (Figure 4(c)), we found that the predominant genera in model rats were as follows: *Phascolarctobacterium*, *Flexispira*, *[Prevotella]*, *Paraprevotella*, *Roseburia*, *Faecalibacterium*, *Bifidobacterium*, *Butyricicoccus*, *Prevotella*, and *Bacteroides*; the abundance of *Oscillospira*, *Desulfovibrio*, *Ruminococcus*, *Shuttleworthia*, *[Ruminococcus]*, *Dorea*, *Collinsella*, *Adlercreutzia*, *Parabacteroides*, *Lactobacillus*, and *Blautia* was markedly decreased. The abundance of the strains mentioned above of SFE treatment group has tended to that of the control group, indicating a meaningful modulation of SFE on gut bacteria. Unweighted_Unifrac-based PCoA (Figure 4(d)) of gut microbiome revealed a clear separation of rats in the model and control groups. After SFE treatment, the gut microbiome of model rats was shown to reverse T2DM-induced structural variations. Thus, we hypothesized that the structure of gut microbiome was significantly changed during the development of T2DM and that SFE played a key role in adjusting gut bacteria of T2DM rats.

#### 3.3.2. Differential Gut Bacteria in T2DM and SFE-Treated Rats

The Linear discriminant analysis (LDA) Effect Size (LEfSe) method was used to identify the bacterial taxa, in which relative abundance varied significantly among the model, SFE treatment, and control rats.

**Figure 5:**
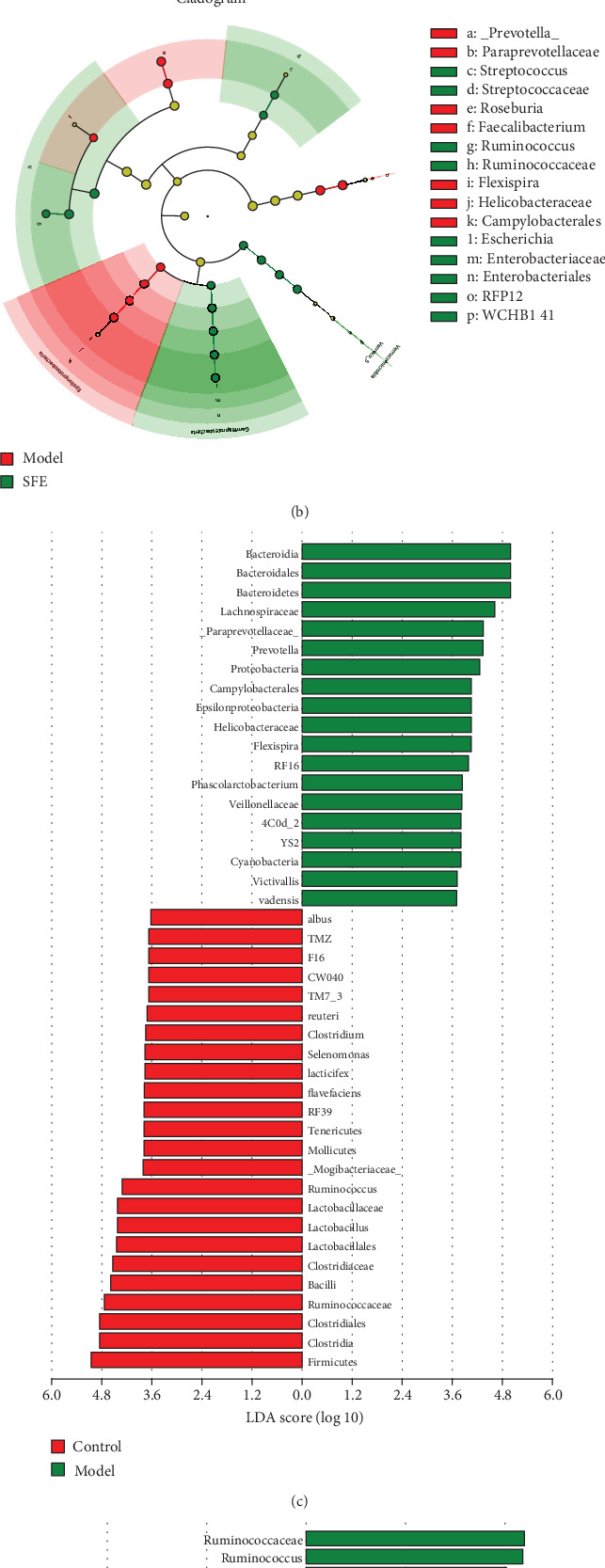
Markedly changed gut bacteria in response to the T2DM and SFE treatments. (a) Cladogram generated by the LEfSe analysis showing discriminative taxa in the eighth week of feces from the control and T2DM groups. (b) Cladogram generated by the LEfSe analysis showing discriminative taxa in the eighth week of feces from the T2DM and SFE groups. (c) LDA scores of discriminative taxa shown in (a). (d) LDA scores of discriminative taxa shown in (b).

Between model and control rats, nineteen bacteria were discriminative in model rats (Figures 5(a) and 5(c)), and between model and SFE-treated rats, fourteen bacteria were discriminative in SFE-treated rats (Figures 5(b) and 5(d)). With the onset of T2DM, colonies including phylum *Bacteroidetes*, *Proteobacteria*, and *Cyanobacteria*, the classes *Bacteroidia*, *Epsilonproteobacteria*, and *4C0d_2*; the order *Bacteroidales* and *Campylobacterales*; the families *Lachnospiraceae*, *Paraprevotellaceae*, *Helicobacteraceae*, *RF16*, *Veillonellaceae*, and *YS2*; the genera *Prevotella*, *Flexispira*, *Phascolarctobacterium*, and *Victivallis*; and the species *vadensis* greatly contributed to differences in the gut microbiome, thereby suggesting that the changes of these bacteria may promote the deterioration of T2DM. After treatment with SFE, the phylum *Verrucomicrobia*; the classes *Verruco_5* and *Gammaproteobacteria*; the orders *WCHB1_41* and *Enterobacteriales*; the families *Ruminococcaceae*, *RFP12*, *Enterobacteriaceae*, and *Streptococcaceae*; the genera *Ruminococcus*, *Escherichia*, and *Streptococcus*, and the species *coli* and *flavefaciens* changed significantly, thereby indicating that these bacteria were associated with an SFE-induced treatment effect in T2DM.

#### 3.3.3. Prediction of the Gut Bacterial Function

**Figure 6:**
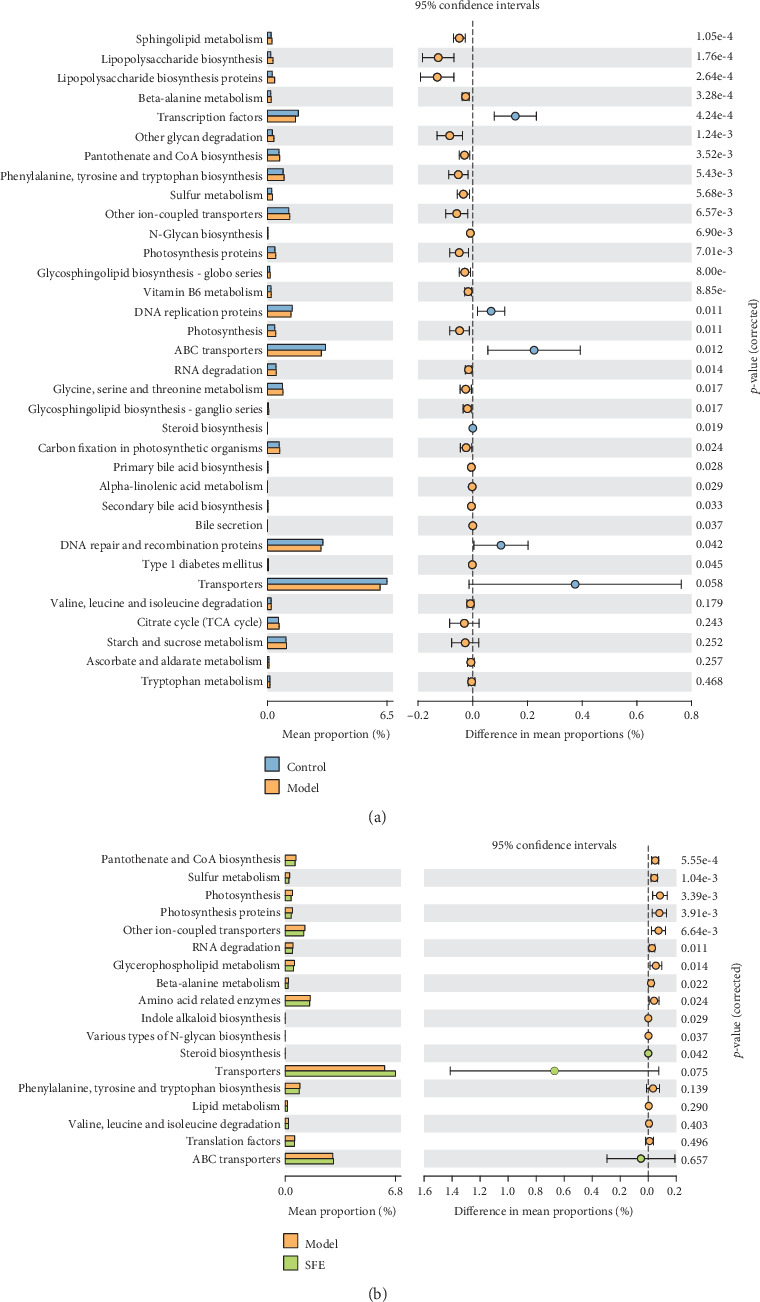
Function prediction of the bacterial community at level III. (a) PICRUSt analysis between the control rats and model rats. (b) PICRUSt analysis between the model rats and SFE treatment rats.

To understand the functional metagenomic profiles of different bacterial communities, we performed PICRUSt analysis based on the KEGG pathway using 16S rRNA data (Figure 6(a) and 6(b)). All functional genes were divided at level III. In the model group, genes relevant to amino acid metabolism, lipid metabolism, energy, carbohydrate, and glycan biosynthesis and metabolism were more abundant, indicating that these metabolic pathways were significantly disturbed in T2DM. It is worth noting that the abundance of genes involved in energy metabolism, lipid metabolism, glycan biosynthesis and metabolism, the metabolism of certain amino acids, and genetic information processing was modified after SFE treatment. Taken together, the results mentioned above suggested that gut bacteria were involved in changes in the systemic metabolic pathway in T2DM and that SFE recovered abnormal metabolic pathways by regulating the abundance of gut bacteria that were involved in host metabolism.

### 3.4. The Results of Fecal Metabolomics Analysis

#### 3.4.1. UHPLC-MS Metabolic Profiles

**Figure 7:**
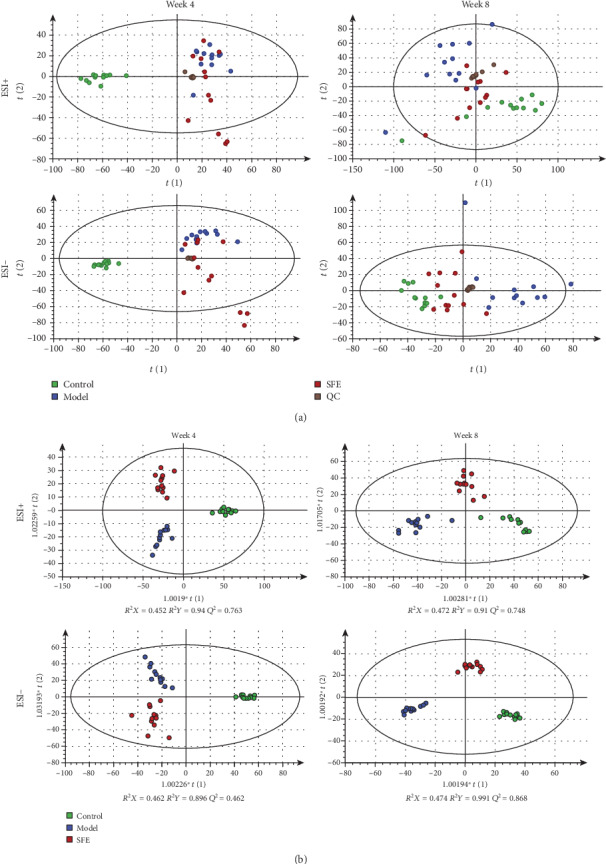
(a) The PCA score plots from the control, model, SFE, and QC groups in the fourth and eighth weeks and (b) the OPLS-DA score plots from the control, model, and SFE groups in the fourth and eighth weeks.

**Figure 8:**
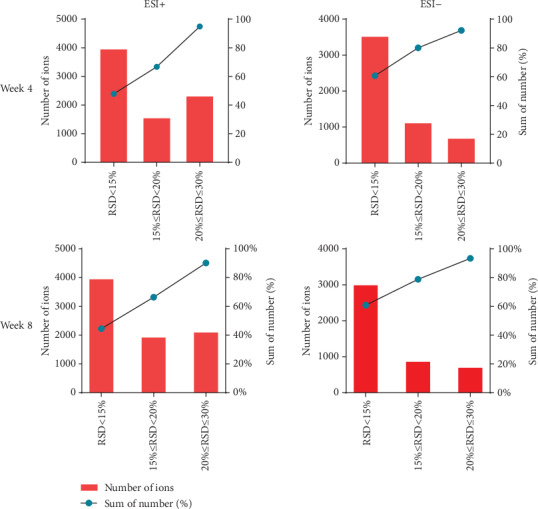
The distribution of RSD in QC samples under positive ion and negative ion modes in the fourth and eighth weeks. The histogram represents the number of compounds in the specified RSD range, and the line graph represents the cumulative percentage of compounds less than the specified RSD.

Base peak chromatograms of feces sample in positive and negative ion mode are presented in Supplementary Materials (Figure S3). QC samples were used to evaluate the reproducibility, stability, and sensitivity of metabolomics analysis; TIC of QC samples in positive and negative ion mode is presented in Supplementary Materials (Figure S4). A high degree of aggregation of QC sample is shown in the PCA score plot (Figure 7(a)), which demonstrated the stability of the system. Furthermore, the results of distribution of RSD in QC samples are presented in Figure 8, and the percentage of compounds with RSD < 30% was greater than 70%; for example, in the eighth week, in positive ion mode, the percentage of compounds with RSD < 15% reached 49.32% and with RSD < 20% reached 73.56%, and in negative ion mode, the percentage of compounds with RSD < 15% reached 65.18% and with RSD < 20% reached 84.41%. Thus, the data showed that the analysis of this method was repeatable and suitable for fecal metabolomics. The PCA was used to study the differences between the control, model, and SFE groups in the fecal metabolomics by an unsupervised statistical method. In the PCA score plot (Figure 7(a)), the separation among the control, model, and SFE groups appeared over time, suggesting a difference among the three groups.

An OPLS-DA model was used to extract potential variations from the control, model, and SFE groups in the fourth and eighth weeks. As depicted in Figure 7(b), the score plots for OPLS-DA presented clear separation among three groups, and the differences between the fourth and eighth weeks could be distinctly identified in OPLS-DA score plots. According to the parameters of the OPLS-DA models, *R*^2^*X*, *R*^2^*Y*, and *Q*^2^ indicated that these models had excellent modeling and predictive ability. The established OPLS-DA models were verified by 200 response permutation testing (RPT). Observing *R*^2^ and *Q*^2^ values and RPT plots that were obtained from the fourth and eighth weeks of all groups, *R*^2^ and *Q*^2^ values produced by permutated Y variables were smaller than the original *Y* variables, the slope of the regression lines was large, and the intercept with the vertical axis was less than zero. These findings indicated that an over-fitting phenomenon did not occur in the original models and that the established models have a good prediction ability. Therefore, subsequent analysis could be performed. For example, the response permutation testing (RPT) plots from the control and model groups in the eighth week are presented in Supplementary Materials (Figure S5).

#### 3.4.2. Potential Biomarker Identification

**Figure 9:**
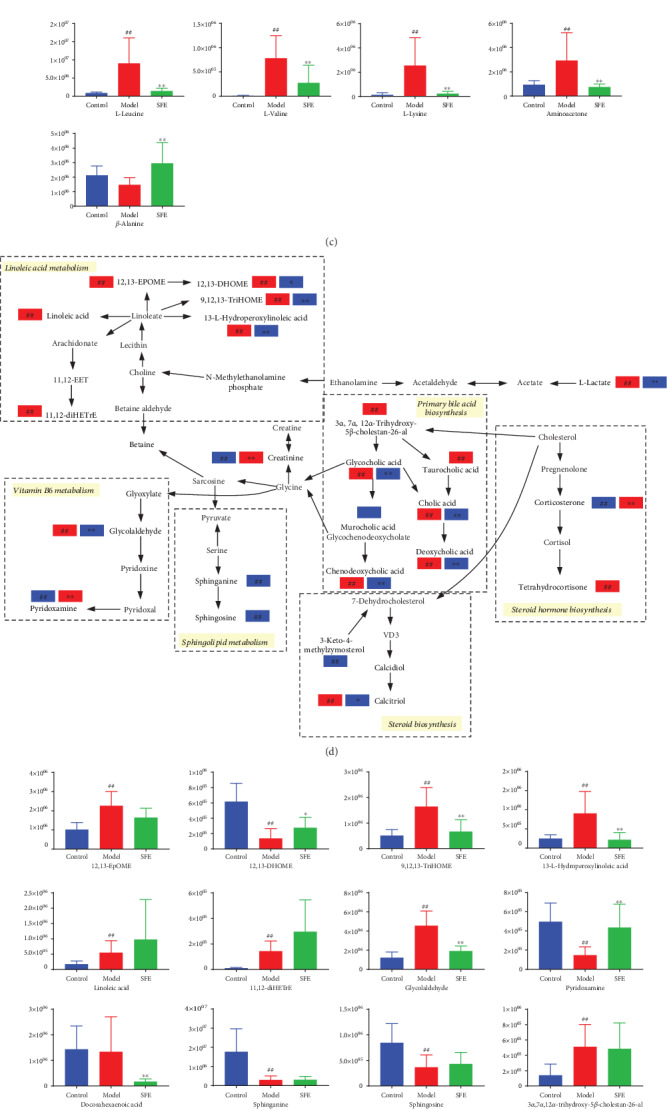
The metabolic pathway analysis. (a) The main metabolic pathways according to the MetaboAnalyst. (b) Amino acid metabolism and (c) the level of metabolites involved in amino acid metabolism. (d) Lipid metabolism and (e) the level of metabolites involved in lipid metabolism. (f) Carbohydrate and nucleotide metabolism and (g) the level of metabolites involved in carbohydrate and nucleotide metabolism. The blue rectangle represents being downregulated in the eighth week, and the red rectangle represents being upregulated in the eighth week. In the expression of the level of metabolites, the ordinate represented the peak area. # indicates a significant change between the control and model groups: ^#^*p* < 0.05, ^##^*p* < 0.01; ∗ indicates a significant change between the model and SFE groups: ^∗^*p* < 0.05, ^∗∗^*p* < 0.01.

Potential biomarkers were selected based on the VIP > 1.0, FC > 2 and <0.5 in the OPLS-DA, and *p* < 0.05 of significant test based on the peak area of variations among different groups. According to the precise molecular masses and necessary MS/MS fragmentations, these potential biomarkers were identified by the HMDB and METLIN database. For example, a potential biomarker with a retention time at 0.94 minutes and a measured mass of 178.0475. Based on this information and the MS/MS fragmentations (Supplementary Materials: Figure S6), this potential biomarker was considered gluconolactone. A total of 61 potential biomarkers were identified in feces samples (Supplementary Materials: Table S2). The changes of biomarkers in different periods were compared according to the peak area and the FC value, and the peak area of potential biomarkers in all groups was expressed as the average ± SD in the form of column in Figure 9.

#### 3.4.3. Metabolic Pathway Analysis

In the present study, MetaboAnalyst was used to link obtained metabolomics data with potential modulation of biochemical pathways [24]. Based on 61 identified biomarkers, the changes in metabolic pathways of T2DM and SFE treatment were filtered out, and 8 metabolic pathways (Figure 9(a)) were selected as the most important metabolic pathways (*p* < 0.05, impact > 0.1) that were related to metabolic disturbances [25]. These included linoleic acid metabolism; valine, leucine, and isoleucine biosynthesis; phenylalanine, tyrosine, and tryptophan biosynthesis; beta-alanine metabolism; phenylalanine metabolism; sphingolipid metabolism; tryptophan metabolism; and arginine and proline metabolism. Metabolic pathways were divided into three major metabolic forms, including amino acid metabolism, lipid metabolism, and carbohydrate and nucleotide metabolism (Figures 9(b), 9(d), and 9(g)), and the levels of potential biomarkers were expressed in the form of column (Figures 9(c), 9(e), and 9(f)).

### 3.5. The Relationship of Feces Metabolites and Gut Microbiota

**Figure 10:**
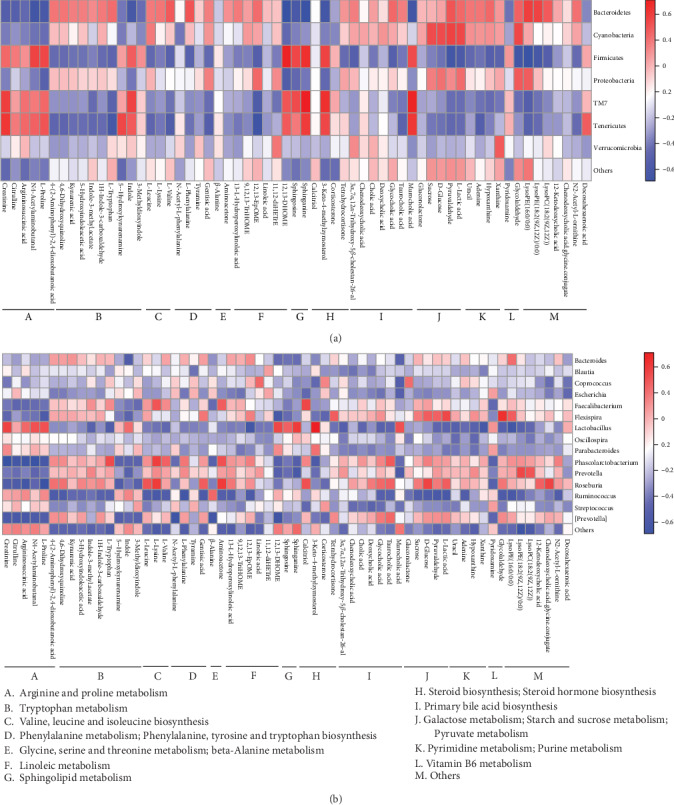
The heat map of correlation analysis between metabolites and gut bacteria at (a) phylum and (b) genus levels.

The OTUs of gut bacteria and the levels of feces metabolites were collected to build a correlation network (Figure 10). The results showed that arginine and proline metabolism, sphingolipid metabolism, steroid hormone biosynthesis, and steroid biosynthesis were positively correlated with *Lactobacillus*, *Oscillospira*, *Parabacteroides*, *Coprococcus*, *Ruminococcus*, and *Streptococcus* and negatively correlated with *Bacteroides*, *Faecalibacterium*, *Flexispira*, *Phascolarctobacterium*, *Prevotella*, *Roseburia*, and *[Prevotella].* The relationship of the remaining metabolic pathways with gut bacteria was opposite to that of the 4 metabolic pathways mentioned above.

## 4. Discussion

In this study, we successfully established T2DM model rats. Model rats showed serious symptoms of T2DM, they significantly lost BW, and FBG, GSP, and the GHb indexes were greatly increased. Additionally, the serum lipid profile was altered and islet cells were severely damaged. A high SFE dose effectively improved BW loss, reduced the FBG level, and recovered the serum lipid profile, GSP, GHb, and pancreatic morphological damage, thereby indicating its effects on alleviating T2DM progression.

The gut microbiota plays a crucial role in the metabolic process. In this study, multiomics was acted as a research tool to determine how T2DM changes gut bacteria and we clarified the interaction between gut bacteria and metabolites. 16S rRNA gene sequencing analysis implied that disturbed composition of gut bacteria may directly aggravate T2DM or indirectly promote the development of T2DM by disturbing amino acids, lipid, energy, carbohydrate, and glycan biosynthesis and metabolism. The metabolomics results demonstrated that T2DM mainly disturbed amino acid, lipid, and glucose metabolism. SFE treatment normalized the structure of gut bacteria and metabolic pathways. Moreover, correlation analysis between gut bacteria and metabolites highlighted their relationship, thereby helping to explain the pathogenesis of T2DM and the mechanism underlying SFE treatment. In this study, we summarized the results of 16S rRNA gene sequencing and metabolomics analysis and discussed the effects of T2DM and SFE on the host-microbial metabolic axis from the relationship between metabolic biomarkers and gut bacteria.

Amino acid metabolism is important to the metabolic network, and different metabolic pathways in amino acid metabolism play the different roles in the development of T2DM. It has long been reported that amino acid metabolism is involved in host-microbiota interactions [6]. Thus, it is of utmost importance to explore the characteristics of amino acids in T2DM.

It has previously been reported that the enhanced metabolism of branched amino acids and aromatic amino acids increased the risk of T2DM and obesities [26, 27], which are positively correlated with insulin resistance (IR) and hyperglycemia [3, 28–30]. In our study, valine, leucine, and isoleucine metabolism; phenylalanine, tyrosine, and tryptophan biosynthesis; and phenylalanine metabolism showed overexpression in the model group according to metabolomics and PICRUSt analysis. *Bacteroides* and *Prevotella* changed markedly with the development of T2DM as indicated by 16S rRNA sequencing analysis. *Bacteroides* and *Prevotella* were the main genera in *Bacteroidetes*, Tong et al. performed a coabundant network analysis and identified two coabundant groups (CAGs) that contained pathogen-like genera *Bacteroides*, and the depletion of these CAGs was associated with glucose homeostasis [31]. These findings were in line with our results that the species *Bacteroides* and *Prevotella* were related to the ferment and biosynthesis of AAA and BCAA and the decrease of these bacteria stimulated the reduction of AAA and BCAA and maintained glucose homeostasis.

Tryptophan metabolism showed overexpression in T2DM according to 16s rRNA sequencing analysis, which was in line with the metabolomics analysis. The tryptophan metabolism has three major pathways and is metabolized into several compounds through the transformation of gut microbiota [32]. The metabolic pathways include the aryl hydrocarbon receptor (AhR) pathway, kynurenine pathway (KP), and serotonin (5-HT) production pathway. Biomarkers in the tryptophan metabolism pathway were metabolized through these pathways. *Escherichia coli* can produce tryptophan, *Lactobacillus*, which is a type of probiotics that links with insulin secretion, can produce AhR ligands, and has high capability to metabolize tryptophan, thereby influencing SCFAs alteration and activating the intestine-brain axis [33, 34]. The production of impaired AhR ligands by microbiota is significant in T2DM. Indole and 3-methyldioxyidole are metabolized from the AhR pathway and transformed by gut microbiota and can produce glucagon-like peptide-1 (GLP-1), which can stimulate the secretion of insulin [35]. The decrease of *Lactobacillus* and the increase of discriminative microbiota (*Escherichia coli*) in the model group was consistent with the decrease of these two metabolites. KP also plays an important role in tryptophan metabolism, and excessive expression of KP was associated with insulin resistance (IR), which can lead to obesity and T2DM [25, 35]; 4-(2-aminophenyl)-2,4-dioxobutanoic acid and kynurenic acid were the metabolites of KP, which were increased in the state of T2DM and decreased after SFE treatment. The 5-HT pathway has a weaker effect than KP in metabolic syndrome, and gut-derived 5-HT is a gastrointestinal signal molecule, which significantly influences intestinal stability and transforms to 5-hydroxyindoleacetic acid (5-HIAA). The increase in 5-HIAA may be attributed to the excessive transformation [35]. Thus, we speculated that excessive transformation may lead to a low level of 5-HT and cause the disordered metabolism of glucose and lipid. SFE relieved enhanced metabolism of AhR, KP, and 5-HT pathways by regulating *Bacteroides*, *Faecalibacterium*, *Flexispira*, *Phascolarctobacterium*, *Prevotella*, *Roseburia*, and *[Prevotella].*

It has previously been reported that alterations in beta-alanine, alanine, aspartate, and glutamate metabolism pathways contributed to the pathogenesis of T2DM [36]. *Ruminococcaceae* was the discriminative bacteria in the SFE group and was considered bacteria that hydrolyze fibers for gut fermentation and played a crucial role in host energy utilization [17, 37]. A decrease in creatinine in model rats represented abnormal energy metabolism and suggested kidney lesions [38], and creatinine was involved in alanine, aspartate, and glutamate metabolism. Thus, we speculated that SFE could rectify the abnormal energy metabolism by regulating *Ruminococcaceae* and alanine, aspartate, and glutamate metabolism. An analysis involving metabolic functional pathways in the T2DM zebrafish microbiome showed that arginine and proline metabolism (KO00330) was downregulated [39] and arginine and its metabolites promoted insulin secretion and improved IR in an obese human. These findings were in line with our metabolomics analysis, and our correlation analysis showed that SFE may upregulate arginine and proline metabolism mainly by decreasing the abundance of *Phascolarctobacterium*, *Prevotella*, *Roseburia*, *Faecalibacterium*, and *Flexispira*.

Lipid metabolism, especially fatty acid (FA) metabolism, is affected by gut bacteria of the host. The oxidation of lipid occurs under oxidative stress, causing proinflammatory effects, thereby resulting in cell damage. Oxidative stress and inflammation can aggravate IR [40]. Therefore, it is warranted to comprehensively analyze lipid metabolism under the metabolization of intestinal bacteria.

Linoleic acid (LA), a type of polyunsaturated fatty acids (PUFA), is part of the *Ω*-6 family (*Ω*-6 PUFA). *Ω*-6 PUFA is generally associated with proinflammatory effects, and increasing evidence confirmed that Ω-6 PUFA can stimulate T2DM [41]. In LA metabolism, LA is converted to arachidonic acid, which can produce 11,12-DHET, promote the release of glucagon, and raise the blood glucose level. Moreover, it can be oxidized to 12,13-EPOME, thereby causing cytotoxicity at high concentrations [42–44]. It has previously been reported that *Roseburia* was positively correlated with LA and *α*-linolenic acid (ALA), which was consistent with our findings [45]. In the metabolomics analysis, LPC and LPE that were involved in glycerophospholipid metabolism were increased in model rats, which was verified by PICRUSt analysis. SFE may exert an antioxidative and anti-inflammatory effect through regulating FAs and glycerophospholipid metabolism with the participation of gut bacteria.

In several studies, it was revealed that increased sphingosine-1-phosphate (S1P) occurs to obese animals and humans, which can lead to IR and overexpression of sphingolipid (SL) metabolism [46]. The sphingolipid metabolism is altered in T2DM and relating to cell death [26]. However, our results obtained from metabolomics analysis were different from those published in some previous studies, which may be due to the following: first, it has been reported that the environment can influence SL levels [47], and most of the previously performed studies were carried out based on biological fluid and tissues, which is different from that used in our study. Second, in the PICRUSt analysis, SL metabolism was significantly upregulated, and SLs and lipopolysaccharide (LPS) were the main components of the membranes of *Bacteroidetes*, and their biosynthesis is related to the development of inflammation in T2DM [30, 48]. We speculated that the increased abundance of *Bacteroidetes* may lead to SL deficiency and cause downregulation of sphingolipid metabolism in fecal metabolomics. Thirdly, sphingosine can synthesize ceramide through ceramide synthase (CerS), and sphinganine can also generate ceramide through enzymatic reaction. Oxidative stress under a high glucose environment will promote inflammatory activation and increase ceramide synthesis. The accumulation of cellular ceramide is related to the pathogenesis of obesity and diabetes. In this study, the downregulation of sphingosine and sphinganine may be related to excessive ceramide conversion induced by oxidative stress [49]. Based on the reasons mentioned above, the different results occurred; however, the underlying mechanism involved should be further investigated.

Overexpression of primary and secondary bile acid biosynthesis and bile secretion in the model group is, according to the PICRUSt analysis, in line with the metabolomics analysis. T2DM can disturb enterohepatic circulation (EHC) and obstruct the reabsorption of bile acids (BAs). BAs can alter the composition of gut bacteria, and conversely, gut bacteria can alter BA. The excessive conjugated primary BAs that cannot be reabsorbed and recycled were metabolized into secondary bile acids by the gut microbiota which can express bile salt hydrolase [50, 51]. If levels of conjugated BAs increase, the loss of tight junction integrity occurs and causes disruption of the intestinal barrier. Gram-positive gut bacteria within the gut lumen are known to express bile salt hydrolase [35, 51, 52], such as *Ruminococcus* and *Streptococcus*, which were found in our study. These bacteria are known to be capable of oxidation and reduction of the hydroxyl groups on the 3-,7-, and 12-carbons of BAs [50]. Overexpression of the primary bile acid metabolism found in our study may attribute to the disrupted EHC and decreased abundance of *Ruminococcus* and *Streptococcus*; SFE may regulate BA metabolism through increasing the abundance of *Ruminococcus* and *Streptococcus* and recovering the reabsorption of BAs.

Carbohydrate and energy metabolism enrichment is common in T2DM. Lactate is a central substance and can be obtained from carbohydrates and transform to glucose; the abnormal level of lactate can imply the disturbance of glucose production [6]. Furthermore, lactate was considered an intermediate in microbial metabolism and can be converted to SCFA by some bacteria, including *Veillonellaceae*, which was the discriminative bacteria in T2DM rats in our study [29]. When compared with model rats, SFE-treated rats showed improved carbohydrate and energy metabolism. Notably, the metabolomics analysis revealed an enriched carbohydrate and energy metabolism, including pyruvate metabolism, galactose metabolism, and starch and sucrose metabolism; however, the PICRUSt analysis implied that the enriched carbohydrate metabolism in model rats was not significant. The possible reason may involve the following: from a micro perspective, *Firmicutes* has been reported to be associated with energy harvest [53], and the species in phylum *Firmicutes*, such as *Ruminococcus* and *Clostridium*, were carbohydrate degrading bacteria [45]. In our study, the decrease of the phylum *Firmicutes* may contribute to insignificant upregulation of carbohydrate metabolism in the model group. From a macro perspective, when insulin secretion was insufficient in T2DM, the entry of glucose into cells was reduced, resulting in a decreased uptake of glucose by the liver, muscle, and other tissues. Therefore, ingested glucose cannot be used by the host and results in excessive consumption. This state is accompanied by impaired glucose oxidation; the overexpression of carbohydrate metabolism occurs to our metabolomics analysis.

## 5. Conclusion

SFE improved the T2DM-related index and inhibited the progress of T2DM. The multiomics approach was successfully applied to reveal that T2DM rats exhibited gut dysbiosis and metabolic disorders and that SFE-treated T2DM through reversing abnormalities of lipid, amino acid, and carbohydrate metabolism and by modulating the interaction between metabolites and the gut microbiome. In summary, in this study, we elucidated the pathogenesis of T2DM and SFE treatment mechanisms from the perspective of the host-microbial metabolic axis systematically.

## Figures and Tables

**Table 1:** Effects of SFE on the serum lipid profile, GHb, and GSP (*n* = 12).

Group	TC (mmol/L)	TG (mmol/L)	HDL-C (mmol/L)	LDL-C (mmol/L)	GHb (%)	GSP (*μ*mol/L)
Control	1.55 ± 0.23^∗∗∗^	0.55 ± 0.07^∗∗∗^	1.32 ± 0.30^∗^	0.42 ± 0.04^∗∗∗^	4.52 ± 0.57^∗∗∗^	154.03 ± 23.04^∗∗∗^
Model	2.40 ± 0.46	1.48 ± 0.60	1.03 ± 0.11	0.75 ± 0.26	13.21 ± 1.56	209.83 ± 15.45
MF	1.86 ± 0.22^∗^	0.60 ± 0.22^∗∗∗^	1.05 ± 0.28	0.41 ± 0.04^∗∗∗^	9.86 ± 1.88^∗∗∗^	189.52 ± 20.34
SFE-L	1.91 ± 0.31^∗^	0.55 ± 0.10^∗∗∗^	1.16 ± 0.20	0.46 ± 0.10^∗∗∗^	11.33 ± 1.01^∗^	178.04 ± 16.27^∗∗^
SFE-H	1.63 ± 0.72^∗∗∗^	0.62 ± 0.07^∗∗∗^	1.18 ± 0.26	0.40 ± 0.14^∗∗∗^	10.89 ± 1.33^∗∗^	174.82 ± 12.76^∗∗∗^

^∗^Significant difference compared with the model group: ^∗^*p* < 0.05; ^∗∗^*p* < 0.01; ^∗∗∗^*p* < 0.001.

## Data Availability

The data used to support the findings of this study are included within the supplementary information files. The original experimental data used to support the findings of this study are available from the corresponding author (Lei Chen chenlei0080@163.com) upon request.

## Supplementary Materials

Supplementary Materials Supplementary 1. 1. Materials and Methods (1.1. Instruments and reagents; 1.2. The quantification of total flavonoid compounds in SFE; 1.3. The conditions of UHPLC-MS analysis of 4 flavonoid compounds in SFE).Supplementary 2. Figure S1: the total ion current (TIC) chromatogram of the flavonoid compounds in SFE. Figure S2: SFE administration altered the gut microbiota structure in T2DM rats. The rarefaction curve of samples from the control, T2DM, and SFE groups performed with (a) Observed species and (b) Shannon. The boxplot of alpha diversity of the control, T2DM, and SFE groups performed with (c) Observed species and (d) Shannon. Figure S3: base peak chromatograms of feces samples in (a) positive and (b) negative ion mode. Figure S4: total ion chromatograms of QC sample in (a) positive and (b) negative ion mode. Figure S5: the response permutation testing (RPT) plots from the model group and control groups in the eighth week. The RPT of OPLS-DA in (a) positive and (b) negative ion mode of feces samples. Figure S6: two-stage tandem mass spectrogram of gluconolactone. Supplementary 3. Table S1: identification of the flavonoid compounds from *Sophora flavescens* EtOAc extract (SFE). Table S2: potential biomarkers identified among the control, model, and SFE treated groups in different weeks.
