# Supplementary material for: An Integrated Fecal Microbiome and Metabolomics in T2DM Rats Reveal Antidiabetes Effects from Host-Microbial Metabolic Axis of EtOAc Extract from Sophora flavescens
Source: Oxid Med Cell Longev. 2020 May 27;2020:1805418. doi: 10.1155/2020/1805418 (PMC7273480; doi:10.1155/2020/1805418)
Supplement: Supplementary Materials — Supplementary 1. 1. Materials and Methods (1.1. Instruments and reagents; 1.2. The quantification of total flavonoid compounds in SFE; 1.3. The conditions of UHPLC-MS analysis of 4 flavonoid compounds in SFE).Supplementary 2. Figure S1: the total ion current (TIC) chromatogram of the flavonoid compounds in SFE. Figure S2: SFE administration altered the gut microbiota structure in T2DM rats. The rarefaction curve of samples from the control, T2DM, and SFE groups performed with (a) Observed species and (b) Shannon. The boxplot of alpha diversity of the control, T2DM, and SFE groups performed with (c) Observed species and (d) Shannon. Figure S3: base peak chromatograms of feces samples in (a) positive and (b) negative ion mode. Figure S4: total ion chromatograms of QC sample in (a) positive and (b) negative ion mode. Figure S5: the response permutation testing (RPT) plots from the model group and control groups in the eighth week. The RPT of OPLS-DA in (a) positive and (b) negative ion mode of feces samples. Figure S6: two-stage tandem mass spectrogram of gluconolactone. Supplementary 3. Table S1: identification of the flavonoid compounds from Sophora flavescens EtOAc extract (SFE). Table S2: potential biomarkers identified among the control, model, and SFE treated groups in different weeks. [file 1805418.f1.pdf]

Supplementary Materials

1. Materials and Methods

1.1. Instruments and reagents

Standard reference norkurarinone, liquiritigenin, calycosin, trifolirhizin, 8-(3-hydroxymethyl-2-butenyl)-5,7,2',4'-tetrahydroxyflavanone, 2' -hydroxy-isoxanthohumol, (2R)-3β,7,4' -trihydroxy-5-methoxy-8-prenylflavanone, (2R)-3 α ,7,4 ' -trihydroxy-5-methoxy-8-prenylflavanone, 3'-hydroxy-daidzein, maackiain, isoxanthohumol, biochanin A, kushenol N, kurarinone, kushenol X, kushenol U, 2'-methoxykurarinone, kushenol L, xanthohumol, kuraridin and norkurarinone (6-lavanduly) were laboratory-made (purity > 98%), isoanhydroicaritin, kushenol I and kushenol E were brought from Chengdu Chroma-Biotechnology Co., Ltd (Chengdu, China), formononetin and isokurarinone were obtained from Dr. Yan-yan Jiang of Beijing University of Chinese Medicine. Analytical grade ethanol, concentrated hydrochloric acid and magnesium powder were obtained from Guangzhou huaxin chemical reagent Co., Ltd (Guangzhou, China). Chromatographic grade acetonitrile and formic acid were purchased from Fisher Scientific Co., Ltd (Cambridge, MA, USA).

The Hitachi U-2000 UV spectrophotometer was manufactured by HITACHI Co., Ltd (Tokyo, Japan). An Ultimate 3000 LC system coupled to a Quadrupole–Exactive Orbitrap–Mass Spectrometry with an ESI source was produced by Thermo Fisher Scientific Co., Ltd (Karlsruhe, Germany).

1.2. The quantification of total flavonoid compounds in SFE

A batch of SFE with 3 samples was weighted. Each sample was dissolved by ethanol. Firstly, the sample solution and norkurarinone standard solution were placed in a test tube that contains magnesium powder, and the test tube was placed in the cold water, and the concentrated HCl was slowly added in it. Secondly, 70 % ethanol was added in the mixture, shaking it well and heating in boiling water for 60 min. Thirdly, cooling the mixture to room temperature, 70 % ethanol was added in it, shaking it well and measuring the absorbance at 483.5 nm.

1.3. The conditions of UHPLC-MS analysis of 4 flavonoid compounds in SFE

The mobile phase consisted of solvent A (0.1 % formic acid in the water, v/v) and solvent B (acetonitrile). The optimal LC parameters were as follows: the flow rate was set at 0.3mL/min, the column temperature was 40 °C, the injection volume was 5 μL. The optimal mass spectrometry conditions were as follow: the scan mode was full scan, the resolution was 70000, the sheath gas flow rate was 15 L/min, the auxiliary gas flow rate was 5 L/min, the spray voltage was 3.0 kV, the capillary temperature was 350 °C, the S-lens RF level was 55.0, the auxiliary gas temperature was 200 °C, the scan range was *m/z* 100-1000.

2. Figure

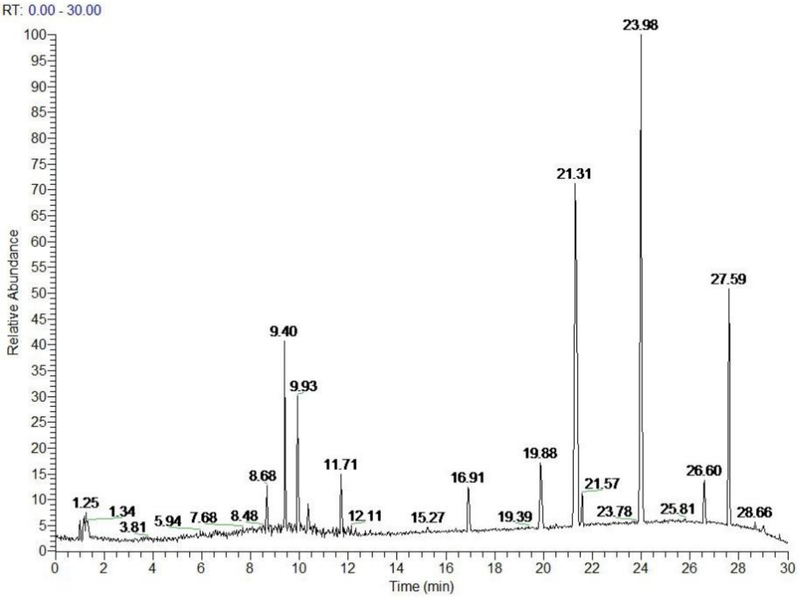

Figure S1: The total ion current (TIC) chromatogram of the flavonoid compounds in SFE.

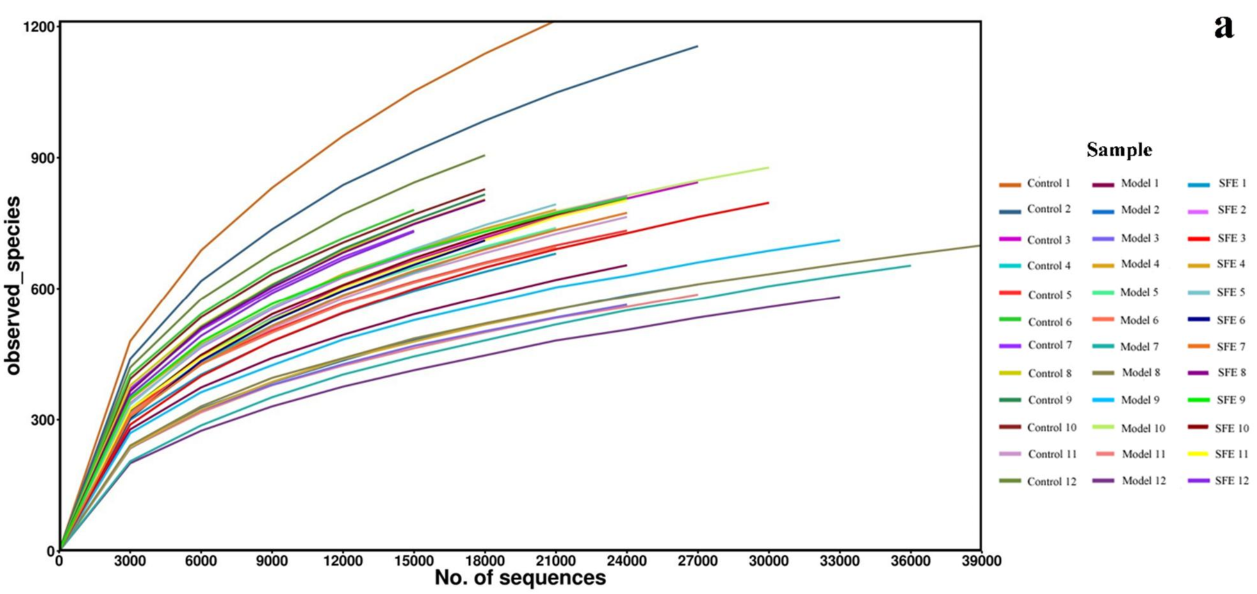

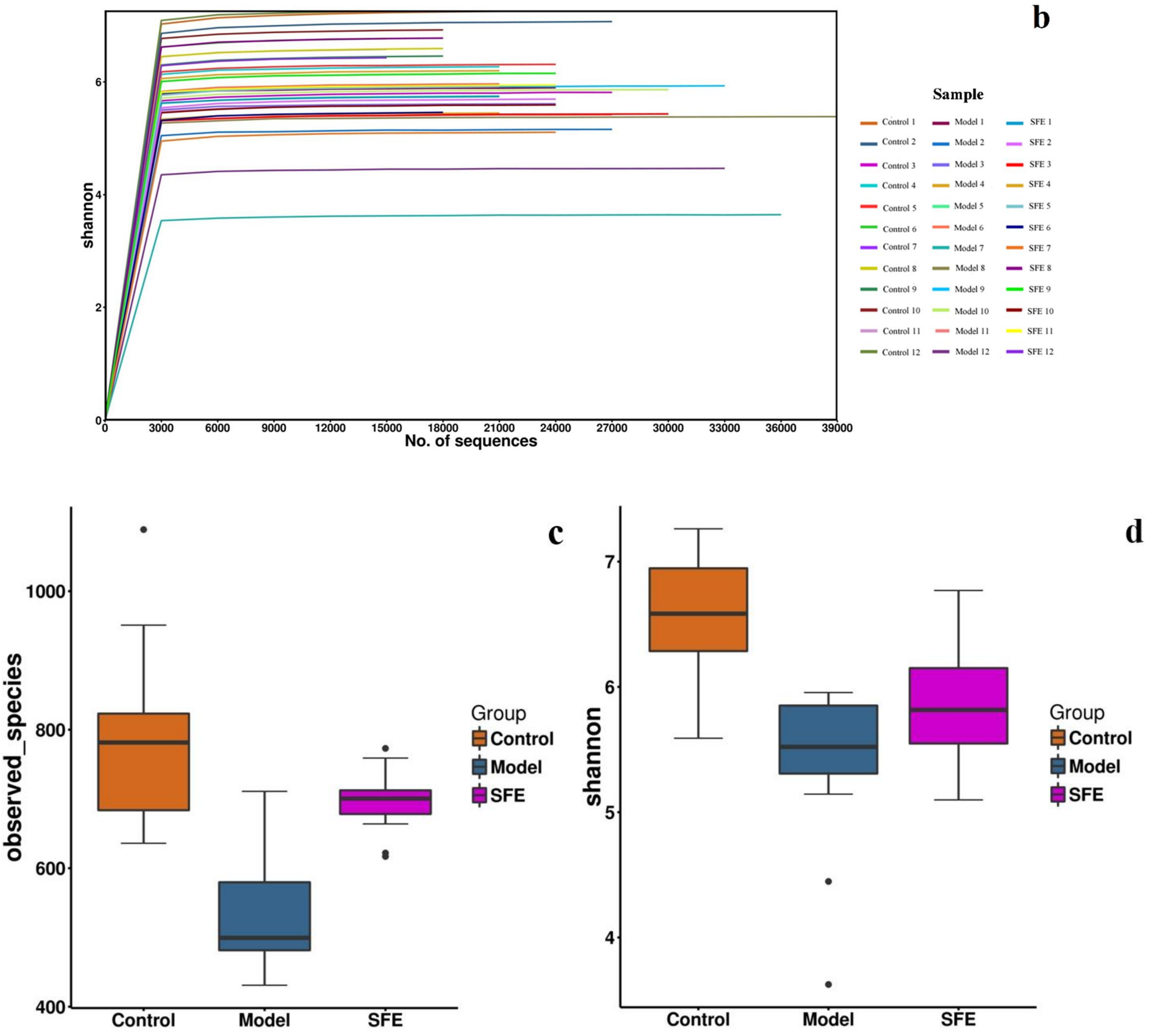

Figure S2: SFE administration altered the gut microbiota structure in T2DM rats. The rarefaction curve of samples from the control, T2DM, and SFE groups perform with (a) observed-species and (b) Shannon. The boxplot of alpha diversity of the control, T2DM, and SFE groups perform with (c) observed-species and (d) Shannon.

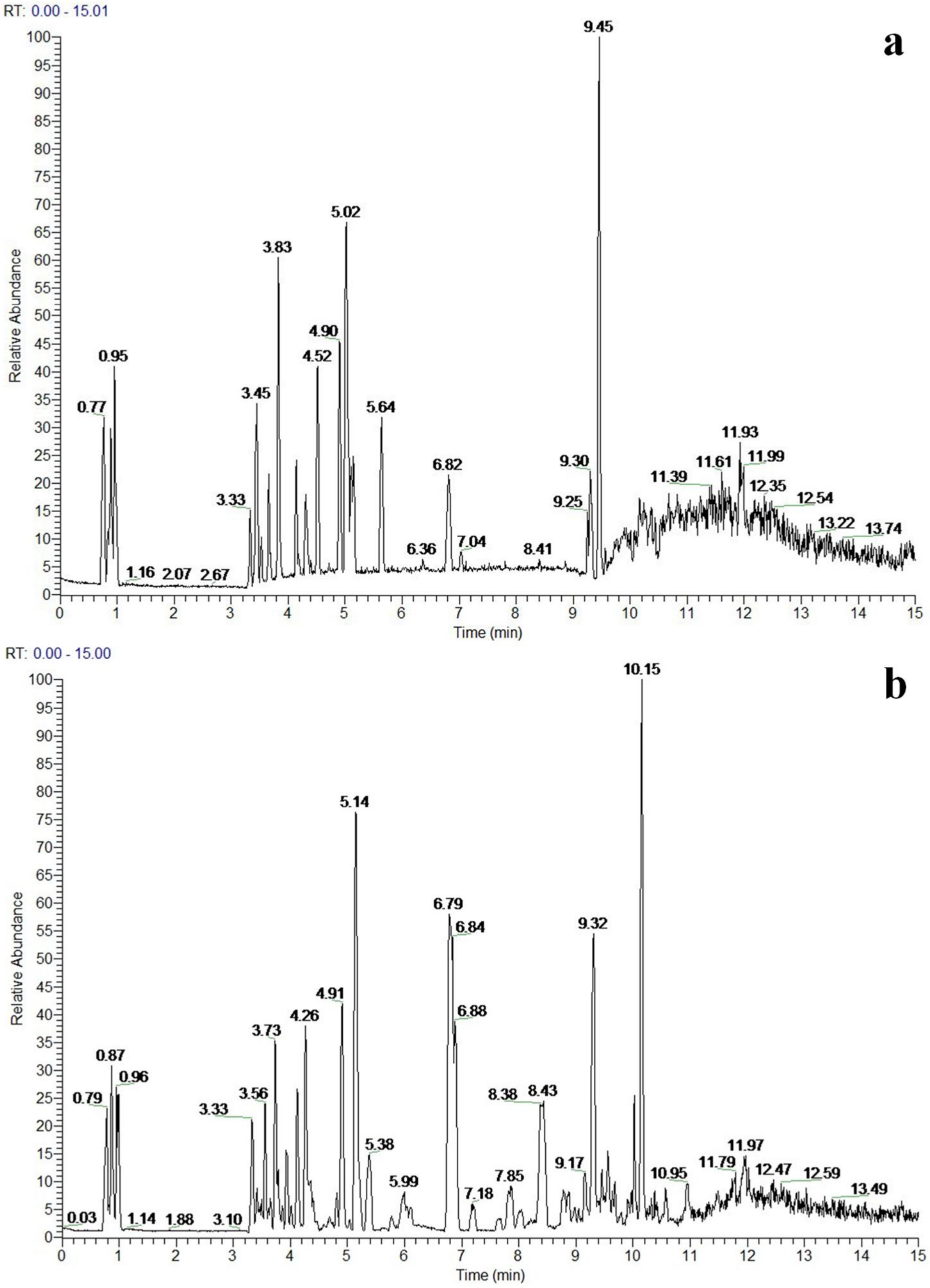

Figure S3: Base peak chromatograms of feces sample in (a) positive and (b) negative ion mode.

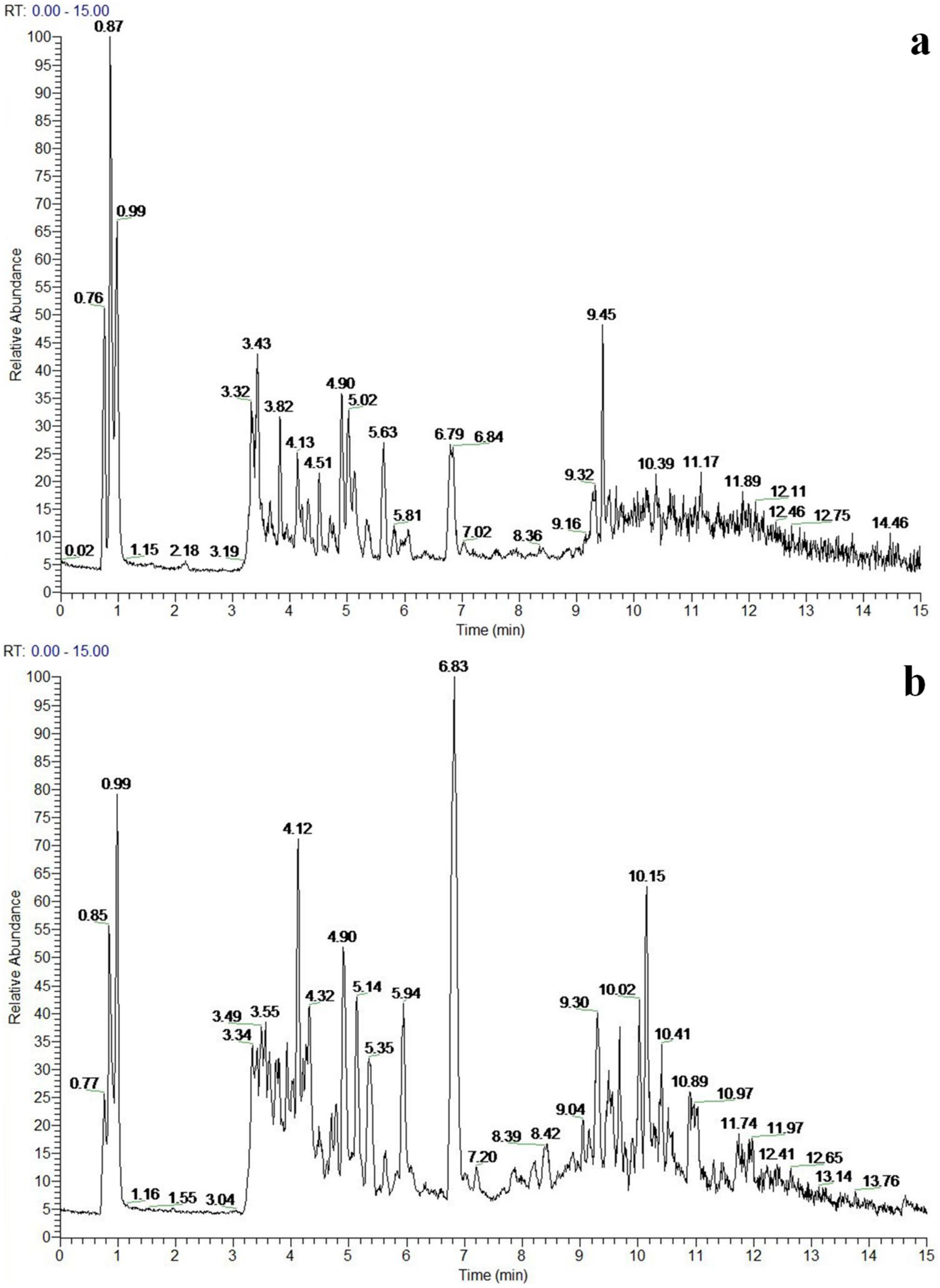

Figure S4: Total ion chromatograms of QC sample in (a) positive and (b) negative ion mode in the eighth week.

a

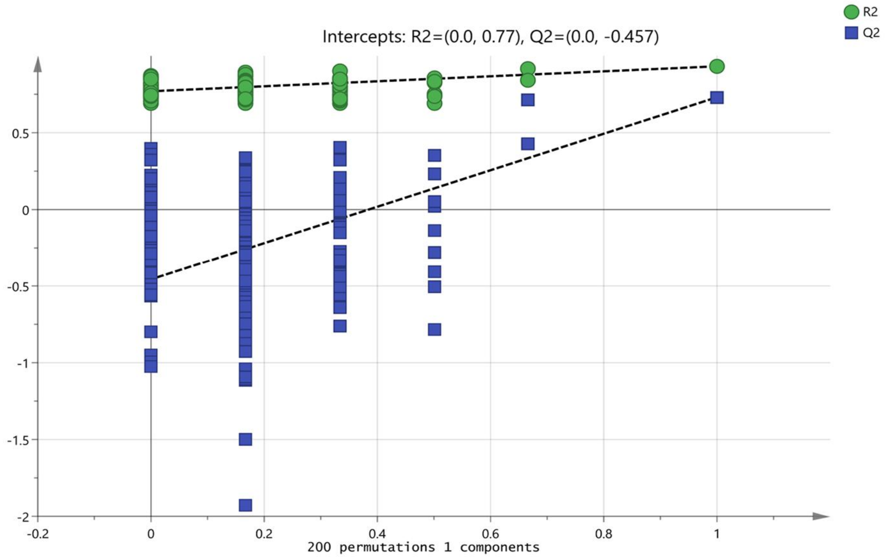

b

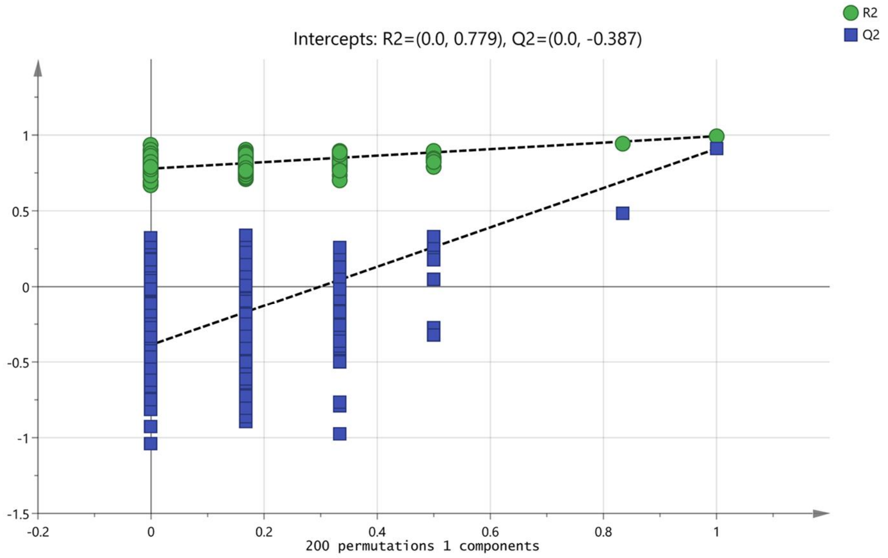

Figure S5. The response permutation testing (RPT) plots from the control and model groups in the eighth week. The RPT of OPLS-DA in (a) positive and (b) negative ion mode of feces samples.

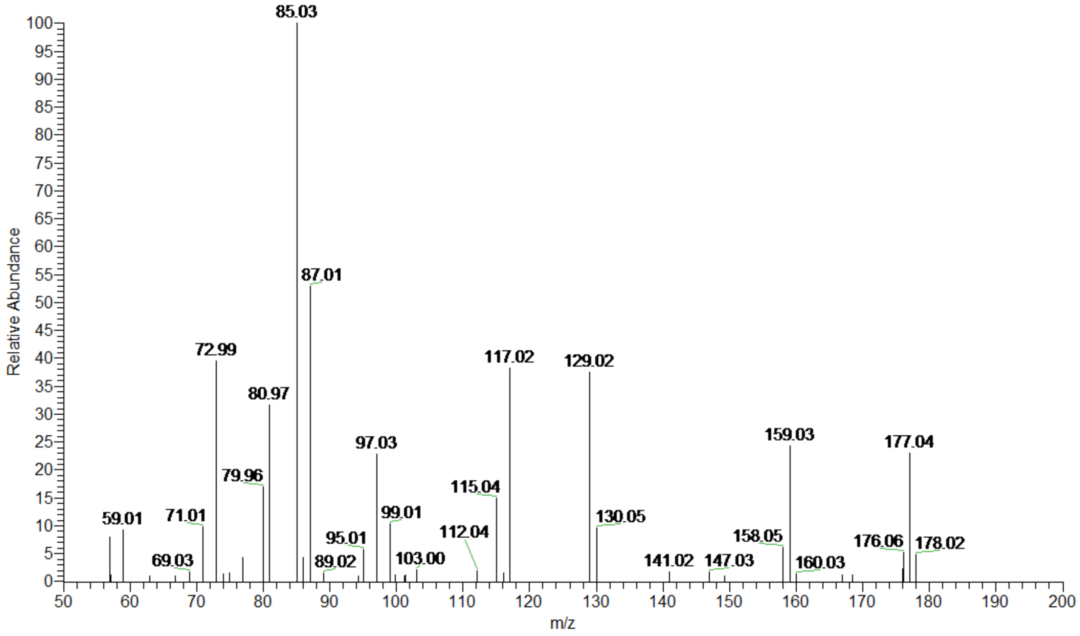

Figure S6: Two-stage tandem mass spectrogram of gluconolactone.

3. Table

Table S1: Identification of the flavonoid compounds from *Sophora flavescens* EtOAc (SFE) extract.

NO	Rt (min)	Mass (*m*/*z*)	MS/MS fragment	Identification
P1	11.01	255.0663	119.0502,135.0088,91.0189,153.0193	Liquiritigenin
P2	11.48	283.0612	268.0375,283.0608,211.0406,239.0344	Calycosin
P3	11.71	491.1195	283.0612,254.0588,255.0653	Trifolirhizin
P4	12.82	269.0455	269.0435,133.0293,201.0555,107.0138	Genistein
P5	13.64	371.1136	209.0819,161.02451,179.0712,124.0165	8-(3-hydroxymethyl-2-butenyl)-5,7,2',4'-tetrahydroxyflavanone
P6	14.57	369.1344	161.0244,207.1026,135.0452,138.0324	2' -hydroxy-isoxanthohumol
P7	15.25	369.1344	207.1026,138.0232,341.1402,147.0244	(2R)-3β,7,4' -Trihydroxy-5-methoxy-8-prenylflavanone
P8	15.27	267.0663	252.0425,223.0395,251.0350,132.0218	Formononetin
P9	15.53	369.1344	207.1025,138.032,341.1384,137.0243	(2R)-3α,7,4' -Trihydroxy-5-methoxy-8-prenylflavanone
P10	16.60	269.0455	269.0455,241.0506,197.0608,183.0452	3'-hydroxy-daidzein
P11	16.60	283.0612	283.0607,255.0659,254.0578,240.0433	Maackiain
P12	16.91	353.1394	119.0502,353.1398,233.0818,175.0037	Isoxanthohumol
P13	17.68	367.1187	367.1186,297.0404,199.0398,253.0501	Isoanhydroicaritin
P14	19.39	283.0612	268.0375,283.0613,239.0347,211.0398	Biochanin A

P15	19.88	453.1919	177.0193,149.0244,275.1650,151.0401	Kushenol N
P16	20.60	453.1919	149.0245,275.1650,177.0193,151.0402	Kushenol I
P17	21.31	437.1969	161.0245,275.1658,137.0241,151.0401	Kurarinone
P18	21.57	439.1762	261.1489,137.0244,177.0194,149.0245	Kushenol X
P19	23.44	421.2020	119.0503,163.0039,301.1442,217.0506	Kushenol U
P20	23.78	451.2126	149.0608,134.0374,163.0037,301.1432	2'-methoxykurarinone
P21	23.98	423.1813	261.1492,161.0244,137.0244,124.0166	Norkurarinone
P22	24.65	439.1762	261.1492,149.0245,177.0193,124.0164	Kushenol L
P23	25.81	353.1394	119.0502,353.1395,233.0813,175.0037	Xanthohumol
P24	26.60	437.1969	437.1968,287.1286,149.0609,163.0035	Isokurarinone
P25	27.00	423.1813	261.1491,161.0244,124.0163,137.0243	Kushenol E
P26	27.58	437.1969	161.0244,137.0244,275.1652,151.0397	Kuraridin
P27	27.59	423.1813	261.1492,137.0245,161.0245,124.0164	Norkurarinone (6-lavanduly)
P28	22.55	385.1293	193.0868,176.0112,191.0349,124.0163	unknown

Table S2: Potential biomarkers identified among the control, model and SFE treated groups in different weeks.

NO	Rt-min	Actual M	Error(ppm)	Formula	Identification	Change trend–4W		Change trend–8W	
						M/C	S/M	M/C	S/M
**ESI+**									
1	0.96	113.0589	3	$C_4H_7NO_3$	Creatinine	↓**		↓**	↑**
2	4.59	117.0578	1	C_8H_7N	Indole	—		↓**	↑**
3	0.92	131.0946	0	$C_6H_{13}NO_2$	L-leucine	—		↑**	↓**
4	0.89	146.1055	0	$C_6H_{14}N_2O_2$	L-lysine	—		↑**	↓**
5	0.86	168.0899	0	$C_8H_{12}N_2O_2$	Pyridoxamine			↓**	↑**
6	0.90	175.0957	0	$C_6H_{13}N_3O_3$	Citrulline			↓**	↑**
7	3.35	180.0899	0	$C_9H_{12}N_2O_2$	5-Hydroxykynurenamine	↓**	—	↓**	↑**
8	3.35	189.0426	0	$C_{10}H_7NO_3$	Kynurenic acid			↑**	↓*
9	3.61	207.0895	0	$C_{11}H_{13}NO_3$	N-Acetyl-L-phenylalanine	↓**		—	↑**
10	3.60	290.1226	13	$C_{10}H_{18}N_4O_6$	Argininosuccinate	↓**		↓**	↑**
11	8.15	346.2144	2	$C_{21}H_{30}O_4$	Corticosterone	↓**		↓**	↑**
12	4.21	312.2301	1	$C_{18}H_{32}O_4$	13-L-Hydroperoxylinoleic acid	↑**		↑**	↓**
13	4.98	416.3290	1	$C_{27}H_{44}O_3$	Calcitriol	↑**	↓**	↑**	↓*
14	0.83	129.0790	0	$C_6H_{11}NO_2$	N4-Acetylaminobutanal	—		↓**	—
15	1.07	137.0841	1	$C_8H_{11}NO$	Tyramine	↓**	—		↑*
16	5.60	280.2402	1	$C_{18}H_{32}O_2$	Linoleic acid	—	↓**	↑**	
17	4.33	328.2402	1	$C_{22}H_{32}O_2$	Docosahexaenoic acid	↑**			↓**
18	7.29	299.2824	1	$C_{18}H_{37}NO_2$	Sphingosine	↑**		↓**	
19	0.89	73.0528	1	C_3H_7NO	Aminoacetone			↑**	↓**

20	3.53	204.0899	0	$C_{11}H_{12}N_2O_2$	L-Tryptophan	—		↑**	
21	4.38	390.2770	2	$C_{24}H_{38}O_4$	12-Ketodeoxycholic acid	↑**		↑**	↓**
22	8.26	477.2855	1	$C_{23}H_{44}NO_7P$	LysoPE (18:2(9Z,12Z)/0:0)			↑**	↓*
23	4.01	189.0790	1	$C_{11}H_{11}NO_2$	Indole-3-methyl acetate	—		↑**	
24	0.90	89.0477	0	$C_3H_7NO_2$	beta-Alanine			—	↑**
25	11.61	396.3392	1	$C_{28}H_{44}O$	3-Keto-4-methylzymosterol			↓**	
26	9.04	301.2981	0	$C_{18}H_{39}NO_2$	Sphinganine			↓**	
27	8.38	519.3325	1	$C_{26}H_{50}NO_7P$	LysoPC (18:2(9Z,12Z))			↑**	
**ESI-**									
28	9.64	434.3396	2	$C_{27}H_{46}O_4$	3a,7a,12a-Trihydroxy-5b-cholestan-26-al			↑**	
29	0.94	178.0477	1	$C_6H_{10}O_6$	Gluconolactone	—		↑**	
30	0.90	112.0273	2	$C_4H_4N_2O_2$	Uracil			↑**	
31	9.46	338.2457	1	$C_{20}H_{34}O_4$	11,12-diHETrE	↓**		↑**	
32	3.02	117.0790	2	$C_5H_{11}NO_2$	L-Valine	↓*		↑**	↓**
33	3.53	163.0633	1	$C_9H_9NO_2$	3-Methyldioxyindole	↓*		↓**	↑**
34	3.46	191.0582	1	$C_{10}H_9NO_3$	5-Hydroxyindoleacetic acid	↑**	—	↑**	↓**
35	4.12	408.2876	2	$C_{24}H_{40}O_5$	Cholic acid	↑**	—	↑**	↓**
36	3.46	145.0528	1	C_9H_7NO	1H-Indole-3-carboxaldehyde	↑**	—	↑**	↓**
37	6.84	392.2927	2	$C_{24}H_{40}O_4$	Deoxycholic acid	↑**		↑**	↓**
38	3.47	165.0790	1	$C_9H_{11}NO_2$	L-Phenylalanine	—		↑**	↓**
39	0.87	342.1162	2	$C_{12}H_{22}O_{11}$	Sucrose	↑**		↑**	↓**
40	3.40	174.1004	2	$C_7H_{14}N_2O_3$	N-Acetylornithine			↑**	↓**
41	0.86	180.0634	1	$C_6H_{12}O_6$	D-Glucose	↑**		↑**	↓**

42	3.49	154.0266	1	$C_7H_6O_4$	Gentisic acid	↑**		↑**	↓**
43	3.35	207.0532	2	$C_{10}H_9NO_4$	4-(2-Aminophenyl)-2,4-dioxobutanoic acid	↑**	—	↑**	↓**
44	3.35	161.0477	2	$C_9H_7NO_2$	4,6-Dihydroxyquinoline	↑**	—	↑**	↓**
45	0.87	60.0211	2	$C_2H_4O_2$	Glycolaldehyde	↑**		↑**	↓**
46	0.87	72.0211	2	$C_3H_4O_2$	Pyruvaldehyde	↑**		↑**	↓**
47	0.87	90.0317	2	$C_3H_6O_3$	L-Lactate	↑**		↑**	↓**
48	4.15	465.3090	2	$C_{26}H_{43}NO_6$	Glycocholic acid	↑**		↑**	↓**
49	10.03	330.2406	1	$C_{18}H_{34}O_5$	9,12,13-TriHOME			↑**	↓**
50	3.78	515.2917	1	$C_{26}H_{45}NO_7S$	Taurocholic acid			↑**	—
51	8.99	296.2351	1	$C_{18}H_{32}O_3$	12,13-EpOME	—	—	↑**	—
52	0.91	135.0545	1	$C_5H_5N_5$	Adenine	—		↑**	—
53	4.93	392.2927	2	$C_{24}H_{40}O_4$	Murocholic acid	↑**		—	↓**
54	5.91	314.2457	1	$C_{18}H_{34}O_4$	12,13-DHOME	↓**		↓**	↑*
55	1.69	115.0633	2	$C_5H_9NO_2$	L-Proline	↓**		↓**	—
56	0.88	136.0385	12	$C_5H_4N_4O$	Hypoxanthine	↑**		↑**	↓*
57	0.99	152.0334	1	$C_5H_4N_4O_2$	Xanthine	↑*		↑**	
58	3.86	449.3141	2	$C_{26}H_{43}NO_5$	Chenodeoxycholic acid glycine conjugate	↑**		↑**	↓**
59	5.40	392.2927	1	$C_{24}H_{40}O_4$	Chenodeoxycholic acid	↑**	—	↑**	↓**
60	9.12	453.2855	1	$C_{21}H_{44}NO_7P$	LysoPE (16:0/0:0)			↑**	↓**
61	4.17	364.2250	1	$C_{21}H_{32}O_5$	Tetrahydrocortisone	—		↑**	—

M, C and SFE indicate the model group, control group and SFE treatment group, respectively. ↑ indicates that the compound is on the rise and ↓ is decrease. A blank indicates that there is no significant difference. *Indicates a significantly different change between the two groups, *$p < 0.05$, **$p<0.01$.
